# Comparing Physics‐Based, Conceptual and Machine‐Learning Models to Predict Groundwater Levels by BMA

**DOI:** 10.1111/gwat.13487

**Published:** 2025-04-21

**Authors:** Thomas Wöhling, Alvaro Oliver Crespo Delgadillo, Moritz Kraft, Anneli Guthke

**Affiliations:** ^1^ Chair of Hydrology Dresden University of Technology (TUD) 01069 Dresden Germany; ^2^ University of Stuttgart, Stuttgart Center for Simulation Science (SC SimTech) 70569 Stuttgart Germany

## Abstract

Groundwater level observations are used as decision variables for aquifer management, often in conjunction with models to provide predictions for operational forecasting. In this study, we compare different model classes for this task: a spatially explicit 3D groundwater flow model (MODFLOW), an eigenmodel, a transfer‐function model, and three machine learning models, namely, multi‐layer perceptron models, long short‐term memory models, and random forest models. The models differ widely in their complexity, input requirements, calibration effort, and run‐times. They are tested on four groundwater level time series from the Wairau Aquifer in New Zealand to investigate the potential of the data‐driven approaches to outperform the MODFLOW model in predicting individual target wells. Further, we wish to reveal whether the MODFLOW model has advantages in predicting all four wells simultaneously because it can use the available information in a physics‐based, integrated manner, or whether structural limitations spoil this effect. Our results demonstrate that data‐driven models with low input requirements and short run‐times are competitive candidates for local groundwater level predictions even for system states that lie outside the calibration data range. There is no “single best” model that performs best in all cases, which motivates ensemble forecasting with different model classes using Bayesian model averaging. The obtained Bayesian model weights clearly favor MODFLOW when targeting all wells simultaneously, even though the competing approaches had the chance to fine‐tune for each tested well individually. This is a remarkable result that strengthens the argument for physics‐based approaches even for seemingly “simple” groundwater level prediction tasks.

## Introduction

Spatially distributed transient groundwater flow models based on partial differential equations (PDEs) as implemented in MODFLOW (Harbaugh 2005), HydroGeoSphere (Therrien et al. 2010), Parflow (Maxwell 2013), or FEFLOW (Diersch 2014), among others, have evolved to the engineering standard for simulating groundwater levels in managed aquifers (e.g., Elshall et al. 2020; Condon et al. 2021). The management of groundwater resources does not consider the complete functioning of the hydrosystem, but rather relies on thresholds set for only a limited number of monitoring wells (Thomann et al. 2020; Bright et al. 2023; Jasechko et al. 2024) which questions the necessity to use data‐hungry and CPU‐time‐excessive PDE‐based models. Even if the main purpose of a model is solely the simulation of groundwater levels at distinct point locations, spatially distributed, PDE‐based groundwater models have been applied to these questions, although simpler models could be equally suited for the task (e.g., Wöhling et al. 2018; Bakker and Schaars 2019).

Predictive accuracy for yet unseen data (potentially extremes) has been a main argument in favor of these mechanistic models in addition to their capabilities for data integration, system understanding, hypothesis testing, and their diagnostic power (Fatichi et al. 2016). Other arguments are that they sustain underlying physical processes, have parameters supposedly pertaining to hydrogeological meaning, and can predict system states that are not part of the calibration data set, for example, groundwater storage or groundwater levels at ungauged locations. However, these models typically require a lot of data for parametrization and specification of space–time dependent model boundaries, have high CPU time demands, and the accuracy of the simulations is susceptible to trade‐offs between calibration targets in combination with potential misspecifications or oversimplifications of model structure.

Thus, several other types and classes of models have been applied—particularly for point‐scale groundwater level predictions—that are computationally much faster, have a lower parametrization effort, and often require less or easier accessible input data. These model classes include simple regression approaches (Sahoo and Jha 2013; Ardana et al. 2022), conceptual models that operate on the level of (linear) storage compartments (Sahuquillo 1983; Zhang and Li 2006; Hong and Wan 2011), transfer‐function noise models (TFN) (von Asmuth et al. 2002, 2008, 2012; Peterson and Western 2014; Zaadnoordijk et al. 2019; Rudolph et al. 2023), Bayesian Networks (Fienen et al. 2013) and a wide range of data‐driven models adapted from the machine‐learning (ML) community (Rajaee et al. 2019; Tao et al. 2022; Boo et al. 2024a). A growing number of studies show the usefulness of these simpler models in the simulation of groundwater time series at a few target points (e.g., von Asmuth et al. 2008; Sahoo and Jha 2013, 2015; Peterson and Western 2014; Zaadnoordijk et al. 2019; Sun et al. 2022; Tao et al. 2022, and others) but there is only a limited number of studies that investigate and compare the performance of models from different categories (Collenteur et al. 2024) particularly in the context of the effort required for the setup, parameterization, and calibration.

A common categorization method of model types is by their degree of complexity or by their physical insight (e.g., Seppelt 2003). PDE‐based models are assumed to give “full” insight in the physics of the system while data‐driven models relate the input to the output with some trained transfer function that does not allow for any insight into the system. The space between PDE‐based and data‐driven models is filled with various categories of hybrids that use both physical concepts (such as storage reservoirs) together with empirical relationships. While this categorization is convenient, model complexity is not simply measured by the degrees of freedom or by the number of parameters (see discussion in Schöniger et al. 2015; Höge et al. 2018). In this context, data‐driven models with a large number of adjustable parameters would also be complex. The degree of physical insight is also not necessarily a good discriminator since insight may also be gained from the trained structure of data‐driven models (see, e.g., Wunsch et al. 2024 for explainable ML). But since this topic is not the focus here, we use the categorization mentioned above and refer to PDE‐based, data‐driven, and hybrid models in this study.

In contrast to the PDE‐based models, hybrid and data‐driven models are typically set up for individual observation wells. This narrows down the calibration target to a single time series that is independent of other calibration constraints that exist in PDE‐based models. This could pose an advantage for hybrid and data‐driven models with respect to the fit to data. Two members of hybrid models that have been applied to simulating groundwater level time series before are eigenmodels (Sahuquillo 1983; Bidwell 2005; Pulido‐Velazquez et al. 2011) and transfer function models (von Asmuth et al. 2008; Collenteur et al. 2019; Zaadnoordijk et al. 2019). Their basic building blocks are storage reservoirs, a concept borrowed from hydrological modeling, which drastically simplifies the (spatial) representation of the groundwater—surface water systems. Another group of methods for model simplification is the dimensionality reduction of the original system by subspace projection (e.g., Siade et al. 2012; Gosses and Wöhling 2019).

The field of data‐driven models currently gets a lot of attention due to the rapid development of ML techniques that have found their way into the field of hydrogeology. Common representatives of ML techniques are simple feed‐forward networks, or Multi‐Layer Perceptrons (MLPs), that connect input to output time series through one or more hidden layers of neurons (Adamowski and Chan 2011; Kouziokas et al. 2018; Wunsch et al. 2018, 2021; Gosses and Wöhling 2019; Afzaal et al. 2020; Sahu et al. 2020), or branching from decision trees, such as random forest (RF) models (Rodriguez‐Galiano et al. 2014; Wang et al. 2018; Yin et al. 2021). They have been advanced to combat vanishing or exploding gradients during their training by regularized weights like in convolutional neural networks (CNNs) (Afzaal et al. 2020; Wunsch et al. 2021), or by including recurrent structures, like in recurrent neural networks (RNNs) (Bowes et al. 2019) or long‐short term memory (LSTM) networks (Bowes et al. 2019; Afzaal et al. 2020; Müller et al. 2021; Wu et al. 2021; Wunsch et al. 2021; Yin et al. 2021). Other types of ML techniques applied in the groundwater level context include support vector machines (SVM) and Gaussian processes regression (GPR), among others (e.g., Sapitang et al. 2021). Recent research has been conducted to further enhance ML models through hyperparameter optimization (Kouziokas et al. 2018; Müller et al. 2021; Wunsch et al. 2022) or the coupling with data imputations such as wavelet transforms (Adamowski and Chan 2011; Zare and Koch 2018; Wu et al. 2021; Boo et al. 2024b). A comprehensive review of recent developments and applications can be found in Boo et al. (2024a); Tao et al. (2022). Many studies report that ML models are computationally fast, are easy to set up, require less data, and often perform well in simulating groundwater time series (Sahoo and Jha 2013; Gosses and Wöhling 2019; Afzaal et al. 2020; Wunsch et al. 2021; Yin et al. 2021; Ardana et al. 2022). Nonetheless, some weaknesses still remain and might limit their applicability for groundwater management, for example issues related to overfitting and generalizability (Rajaee et al. 2019) as well as their ability to extrapolate under different model forcings and extremes (Murphy 2012).

Given the large variety of models from different modeling classes, practitioners face the challenge of model choice. For alternative models, it is typically based on model performance, that is, the ability to fit historic data. The metric to quantify model fit has an impact on model choice, as well as we will analyze and demonstrate in this study. Further, if alternative models are tested, they are typically from one model class, and different model classes are rarely tested together. Several studies have compared different ML techniques (e.g., Rajaee et al. 2019; Afzaal et al. 2020; Wunsch et al. 2021) and reported mixed results. A recent study by Collenteur et al. (2019) analyzed several data‐driven techniques and lumped parameter models and also found that no model outperformed all others. Only a few studies compared PDE‐based models and ML techniques and found the data‐driven models competitive with respect to groundwater level predictions (Moghaddam et al. 2019; Chen et al. 2020; Yin et al. 2021). We extend this work by testing members from all three model classes together. In addition to evaluating and ranking the performance of individual models, we test the extrapolation skill of the models with extreme data that is outside the calibration range. This study also explores the benefits of model ensemble simulations (model averaging) using ensembles composed of pre‐calibrated realizations or post‐calibration ensembles of the individual models (i.e., an ensemble of ensembles). Utilizing Bayesian model selection (BMS), we provide an objective framework for model choice that can be applied to model ensembles containing members from any model type and any model class.

## Materials and Methods

The data used for this model comparison study is introduced first. Then, a short description of the six models and modeling strategies tested follows. We have included models of different complexity and type, some of which are more commonly applied for predicting groundwater levels than others. The models included in our study are a transient, spatially explicit 3D MODFLOW flow model (MOD), a 2D eigenmodel approach (EM), a transfer‐function‐noise (TFN) model, and three machine learning (ML) techniques: a multi‐layer perceptron model (MLP), a LSTM model, and a RF model. Next is the description of our procedure for model evaluation. Finally, the framework for ensemble modeling, Bayesian model averaging (BMA) and selection (BMS) is outlined.

### Data

The data for this study was drawn from the unconfined Wairau Aquifer in the coastal region of the Marlborough District of New Zealand. The aquifer exemplifies many heavily managed coastal aquifers in New Zealand and elsewhere. The region has been intensively studied before (Davidson and Wilson 2011; Raiber et al. 2012; Wilson and Wöhling 2015; Wöhling et al. 2020) and models of different complexity have been set up in the past (Wöhling et al. 2018; Gosses and Wöhling 2019; Wöhling et al. 2020; Wöhling and Burbery 2020; Ejaz et al. 2022). First, we briefly summarize the study area; then, we present the groundwater level data used for the analysis, and finally, we describe the input data required as model forcings by the individual models.

#### Study Area

**Figure 1:**
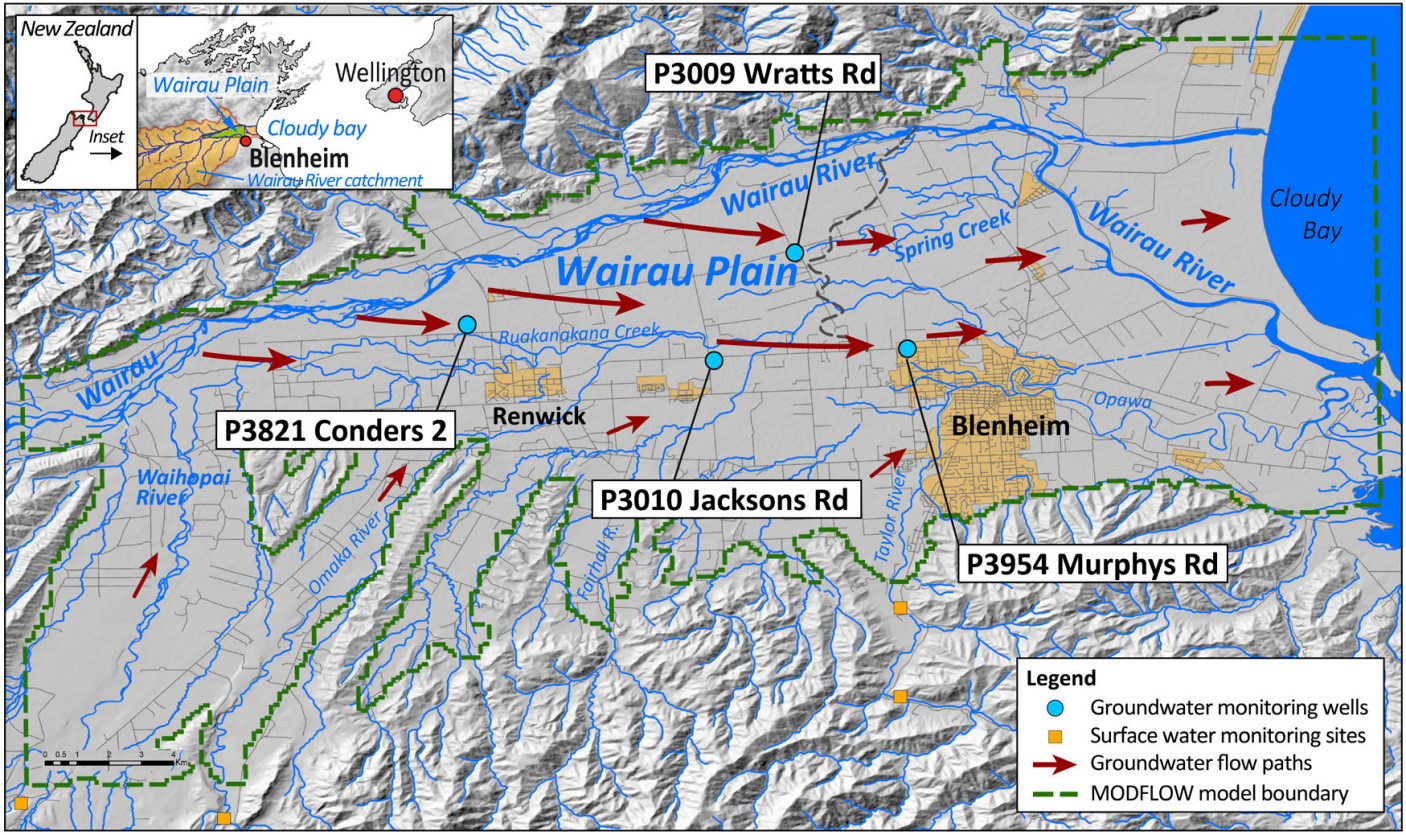
The Wairau Plain study site. The locations of the groundwater monitoring wells investigated in this study are identified by blue dots. The MODFLOW model domain is marked by the green dashed line.

The Wairau Aquifer consists of unconfined, highly transmissive gravels from the Holocene period which get increasingly confined by a wedge of fine‐textured marine sediments at the coast (e.g., Brown 1981; MCB 1987; Raiber et al. 2012; Wilson and Wöhling 2015; Morgenstern et al. 2019; Wöhling 2019; Wöhling et al. 2020). The aquifer is mainly recharged by the Wairau River which is the largest gravel‐bed river in the Marlborough District of New Zealand. Before the river discharges into Cloudy Bay, it flows across the Wairau Plain, where it is initially perched above the regional water table (in the west) and then increasingly connected to the groundwater in the coastal region in the east (Figure 1). At the interface between unconfined and confined gravels, groundwater emerges at the land surface as a series of springs. Groundwater is abstracted from the Wairau Aquifer for vineyard irrigation (the primary land‐use at the Plain), as well as industrial and municipal water uses. The climate of the Wairau Plains is temperate with a mean temperature of 13.2 °C, mean annual rainfall of approximately 640 mm, and mean annual potential evapotranspiration of 1025 mm (MRC 2025). The estimated contribution of land surface recharge to the total recharge is small (<6%) compared to the river recharge (Wöhling et al. 2020). Further details about the study area have been presented in Davidson and Wilson (2011); Wilson and Wöhling et al. (2015); Wöhling et al. (2018, 2020) and are therefore not repeated here.

#### Groundwater Levels

The Marlborough District Council (MDC) operates an environmental observation network that includes groundwater monitoring wells with thresholds that are written in the Marlborough Environment Plan (MEP) for management purposes. We used the records of four monitoring wells as benchmark records in our study (Figure 1). One well (P3821) is located in the upper region of the Wairau Plain, one well is close to the spring region (P3009), one well is further away from the river at the southern margin of the aquifer (P3010) and one well lies in the margins of the confining sediments (P3954). The groundwater levels in the unconfined parts of the Wairau Aquifer exhibit a strong seasonality and get increasingly damped with both increasing confinement and increasing distance from the river (Figure 1). Daily values of groundwater level data for the time period 2000 to 2023 was obtained from the MDC.

#### Model Forcings

Models require model inputs—aka model forcings—to run. The different models tested in this study have different input requirements which are presented in Table 1 and summarized here. The meteorological inputs are daily values of precipitation *P*, potential evapotranspiration *ET*_0_, and air temperature *T*. This data was both sourced as station data from the Marlborough Research Centre in Blenheim, subsequently denoted with hyperscript (·)^BRC^, and as gridded data from the Virtual Climate Station Network (VCSN) by the National Institute of Water and Atmosphere (NIWA) (Tait et al. 2006) which is subsequently denoted as (·)^VCSN^. Land surface recharge *Q*_LSR_ and groundwater abstraction matching the irrigation demand of the viticulture *Q*_Irr_ was estimated by a previously developed, grid‐based land‐surface model (Wöhling et al. 2018). Daily mean values of Wairau River flow *Q*_riv_ at the Barnetts Bank gauging station near SH1 (Figure 1) and groundwater pumping data *Q*_abs_ for municipal and industrial uses were provided by the MDC.

**Table 1:** Model Specification Overview

Model	Parameter Number	Input Requirements	Number of Realizations	Calibration Period	Evaluation Period
MODFLOW (MOD)	526	$P{\left(t,x,y\right)}^{\text{VCSN}}$, ${\mathrm{ET}}_{0}{\left(t,x,y\right)}^{\text{VCSN}}$, $Q_{\mathrm{riv}}\left(t\right)$, $Q_{\mathrm{abs}}\left(t,x,y\right)$	1001	February 1, 2014 to February 20, 2017	January 25, 2018 to December 31, 2023
Eigenmodel (EM)	10	$P{\left(t\right)}^{\mathrm{BRC}}$, ${\mathrm{ET}}_{0}{\left(t\right)}^{\mathrm{BRC}}$, $Q_{\mathrm{riv}}\left(t\right)$, $Q_{\mathrm{abs}}\left(t\right)$	P3821: 6405 P3010: 4770 P3009: 6489 P3954: 6909	January 1, 2015 to January 24, 2018	January 25, 2018 to December 31, 2023
TFN	4–10	Different combinations of: $P{\left(t\right)}^{\mathrm{BRC}}$, $T{\left(t\right)}^{\mathrm{BRC}}$, $Q_{\mathrm{riv}}\left(t\right)$	7	January 1, 2002 to January 24, 2018	January 25, 2018 to December 31, 2023
MLP	7		14		
LSTM	8		14		
RF	9		14		

### Models

#### MODFLOW Model (MOD)

A detailed transient surface water—3D groundwater flow model (MODFLOW‐NWT, Niswonger et al. 2011) has been previously set up for the recharge section of the Wairau Aquifer by Wöhling et al. (2018). Spatially, it has been extended to the east, south, and west to better represent model boundaries and allow a more explicit exchange with the various rivers and streams in the area. The hydrogeology of the Wairau Plains has been investigated in detail by Brown (1981); Basher et al. (1995); Davidson and Wilson (2011); Raiber et al. (2012); Wilson (2016), among others, and is therefore not repeated here.

Time‐variant boundary conditions considered in the model are the Wairau River, low‐land springs, certain variable head boundaries, as well as land‐surface recharge and groundwater abstraction. The MODFLOW model possesses a total number of 526 parameters that were calibrated for the time period February 2014 to February 2017 using the PEST software (Doherty 2016a), different data types (groundwater levels, spring flows, differential river flow gauging) at various locations, as well as soft targets derived from expert knowledge. To increase the robustness of the parameter estimates and the efficiency in calibration, PEST tools for regularization and subspace projection techniques were applied (Doherty 2003, 2016b). More detail about the model conceptualization and setup, parametrization, and calibration can be found in the Supporting Information for this paper, as well as in Wöhling et al. (2018); Wöhling (2019); Wöhling et al. (2020).

After model calibration, post‐calibration predictive uncertainty was estimated by generating an ensemble of 1000 null‐space Monte Carlo (NSMC) samples with the PEST suite of tools (Tonkin and Doherty 2009; Doherty et al. 2010; Doherty 2016b). The MOD ensemble of 1001 model realizations is then used in our further analysis.

#### Eigenmodel (EM)

Eigenmodels massively reduce the dimensionality of 3D spatially explicit groundwater flow models and thus are computationally cheap while maintaining some physical meaning of the considered groundwater system. Eigenmodels represent the groundwater system as a set of linear reservoirs arranged in series along a vertical slice of an aquifer with homogeneous and isotropic hydraulic properties. Groundwater levels are computed by the eigenvalue solution to the transient groundwater flow problem (Sahuquillo 1983) for perfectly connected streams (Pulido‐Velazquez et al. 2005; Bidwell et al. 2008). The eigenmodels used in this study have been set up previously by Wöhling and Burbery (2020) for the operational forecasting tool AquiferWatch. The eigenmodels require inputs of $P{\left(t\right)}^{\mathrm{BRC}}$, ${\mathrm{ET}}_{0}{\left(t\right)}^{\mathrm{BRC}}$, $Q_{\mathrm{riv}}\left(t\right)$, and $Q_{\mathrm{abs}}\left(t\right)$. Land‐surface recharge, river recharge, and irrigation abstraction were lumped over the domain and computed by a soil–water balance model. Post‐calibration parameter ensembles for individual eigenmodels corresponding to the 4 monitoring wells were derived using Markov chain Monte Carlo (MCMC) simulation and the time period January 1, 2015 to January 25, 2018 as described in Wöhling and Burbery (2020). The corresponding four eigenmodel ensembles of simulated groundwater levels were then used for further analysis in this study. More details about eigenmodels can be found in the Supporting Information and in the paper by Wöhling and Burbery (2020). A summary of model inputs and the number of parameters can be found in Table 1.

#### Transfer‐Function Noise Model

TFN models relate input time series to output time series through a statistical model (von Asmuth et al. 2002). A common approach for the definition of this statistical model in the field of groundwater modeling has been the convolution of input time series with given impulse response functions to generate the output time series of interest (von Asmuth et al. 2002). Different response functions can be utilized for the varying inputs of the TFN model to adequately describe the relationship to the output (von Asmuth et al. 2008; Collenteur et al. 2019). The linear TFN models applied in this study were set up with the Pastas Python package (Collenteur et al. 2019) and differ in their inputs used, with seven distinct input combinations of $P{\left(t\right)}^{\mathrm{BRC}}$, $T{\left(t\right)}^{\mathrm{BRC}}$, $Q_{\mathrm{riv}}\left(t\right)$, $Q_{\mathrm{riv}}\left(t\right)$ and $T{\left(t\right)}^{\mathrm{BRC}}$, $P{\left(t\right)}^{\mathrm{BRC}}$ and $T{\left(t\right)}^{\mathrm{BRC}}$, $P{\left(t\right)}^{\mathrm{BRC}}$ and $Q_{\mathrm{riv}}\left(t\right)$, $P{\left(t\right)}^{\mathrm{BRC}}$ and $T{\left(t\right)}^{\mathrm{BRC}}$ and $Q_{\mathrm{riv}}\left(t\right)$, respectively. For the different input types, gamma response functions were chosen for $P{\left(t\right)}^{\mathrm{BRC}}$ and $T{\left(t\right)}^{\mathrm{BRC}}$, while the polder response function was chosen for the $Q_{\mathrm{riv}}\left(t\right)$ input time series. See the Supporting Information for more information. The first 80% of the total time series were used for model calibration (Table 1) with the standard settings of Pastas in regard to optimization options.

#### Multi‐Layer Perceptron

MLPs are one of the more common ML models applied to time series modeling and have been widely used in groundwater modeling (Daliakopoulos et al. 2005; Adamowski and Chan 2011; Sahoo and Jha 2013; Wunsch et al. 2018; Gosses and Wöhling 2019; Rajaee et al. 2019; Sahu et al. 2020; Ardana et al. 2022). MLP's feed input time series (the “input layer”) through a set of hidden layers, consisting of individual nodes with varying weights and biases, to generate an output time series (the “output layer”) (Murphy 2012). MLP and other ML models have internal settings related to the model structure such as the layer size, window size, and the number of nodes. These model settings are termed hyperparameters. Due to the high speed of the model calibration, where the weights and biases of the hidden nodes are adjusted to optimize model fit, some hyperparameters are often varied or optimized as well (Daliakopoulos et al. 2005; Kouziokas et al. 2018; Wunsch et al. 2018, 2021, 2022; Müller et al. 2021). The MLP models in this study were set up in the Python programming suite utilizing the Keras and Tensorflow (Abadi et al. 2015) packages. Inputs of precipitation, temperature, and Wairau River flow were used in all seven possible input combinations and with two window sizes, respectively (see Supporting Information for more details). Hyperparameters were partially optimized by grid search and partially kept constant to values estimated during preliminary tests.

The best‐fitting model for each of the input and window size combinations was chosen for the following analysis, resulting in the 14 final MLP models used in this study. The overall time series were split into training and testing data equal to 80% and 20%, respectively, resulting in the calibration and validation time periods listed in Table 1.

#### Long Short‐Term Memory Networks (LSTM)

LSTM models are a special form of recurrent models (Hochreiter and Schmidhuber 1997) with a set of hidden layers that relate the input time series to the output time series through adjustable weights and biases of individual nodes, in a similar way to MLP models. Through the addition of structures called gates, which govern the retaining, updating, or forgetting of past information, LSTM models are especially suited for time‐series simulation and have been recently applied in groundwater modeling as well (Bowes et al. 2019; Afzaal et al. 2020; Müller et al. 2021; Wu et al. 2021; Wunsch et al. 2021; Yin et al. 2021). Similar to the MLP models, the LSTM models were set up with Keras and Tensorflow in Python. Identical input combinations to the MLP models were used, and similarly, hyperparameters were partially fixed, partially varied (c.f. the Supporting Information). Splitting of the time series in training and testing periods was done in identical fashion as for the MLP models (Table 1).

#### Random Forest Model

RF models are an ensemble technique that combines multiple independent decision trees by averaging their outputs, first introduced by Breiman (2001). They keep the simplicity and interpretability of decision trees while being more robust (Hamza and Larocque 2005) and have been applied to many regression problems in hydrology and groundwater research over the last decades (Loos and Elsenbeer 2011; Booker and Snelder 2012; Rodriguez‐Galiano et al. 2014; Li et al. 2016; Rahmati et al. 2016; Wang et al. 2018; Yin et al. 2021). The RF models in this study were set up using the Scikit‐Learn Python package (Pedregosa et al. 2011). In accordance to the MLP and LSTM setups described above, the same input combinations and window sizes were chosen to create 14 RF models, again with partially variable and partially fixed hyperparameters and scaled data time series (c.f. the Supporting Information).

### Evaluation of Individual Models

As described above, each model was calibrated with its own input data and calibration period. Thus, the models have not been given exactly the same amount of information in model calibration. This setup, however, is very realistic in practical applications and does not restrict us from evaluating and comparing their predictive performance. ML and hybrid models are often trained on as much data as is available because they tend to overfit when given too little data. MODFLOW, on the other hand, is calibrated on less data due to run‐time restrictions and often limited data availability. But it is generally accepted that a shorter calibration period is a disadvantage for models that need to predict system states that fall outside of the calibration period.

In this study, we didn't focus on model performance in calibration; instead, the performance of the individual models was evaluated for a common time period of independent data that was not used in calibration, which we call “evaluation period” in the following. The performance was evaluated for the time period January 25, 2018 to December 31, 2023 (Table 1) separately for each of the four wells in this study. The evaluation criteria applied here were the root mean squared error (RMSE), the coefficient of determination (*R* ^2^) and the (mean) bias as described in Wöhling et al. (2013), as well as the Kling‐Gupta efficiency (KGE, Gupta et al. 2009). Please refer to the Supporting Information on the definition of these criteria. A perfect fit of the model simulations to the data would be characterized by RMSE and bias values of zero as well as *R* ^2^ and KGE values of unity.

The calibration of the six model types for the four monitoring wells resulted in 6 × 4 model ensembles (of different size) that were used in our analysis. The individual members of a model ensemble are called realizations in the context of this study. In a first step, we identified and evaluated the model realizations with the best fit to the data in the evaluation period. The choice of the evaluation criteria to determine the “best” model among the set of candidate models (model realizations) is largely subjective and reflects the modelers choice to prefer particular aspects of the data. Different criteria will identify different realizations as being the best one and thus have an impact on how models in a model ensemble are ranked. Please refer to the discussion in Wöhling et al. (2013) for further details on the choice of optimization criteria. We analyzed the impact of the performance‐metric choice on model ranking by evaluating models using (1) the RMSE and (2) the KGE. The RMSE is related to the sum squared error and equivalent to the Gaussian likelihood measure applied in the BMA scheme (Equation 6) which is used as a more objective model ranking (see discussion below). The KGE is a criterion commonly used in hydrology to evaluate time‐series data and is a composite of linear correlation, relative variability and normalized bias (Gupta et al. 2009; Wöhling et al. 2013). The “acceptable” range of KGE values and its variants is a matter of debate (Knoben et al. 2019; Towner et al. 2019; Althoff and Rodrigues 2021; Cinkus et al. 2023), but it can be easily shown that a model that generates the (time‐invariant) mean of the observations, obtains a value of KGE = −0.41. An acceptable model should, however, obtain larger values than this. A range of KGE ≧0.5 and ≧0.75 is accepted by some studies as “intermediate” and “good,” respectively, (Thiemig et al. 2013; Towner et al. 2019) which is also adopted here.

### Multi‐Model Analysis

In a second analysis step, we combined all six model ensembles for each of the monitoring wells to four multi‐model ensembles. Then we evaluated the performance of the six models using Bayesian model averaging (BMA) and selection techniques (again, only for the evaluation period). BMA was applied here to determine the relative performance of individual model ensembles to simulate groundwater levels for each of the monitoring wells in the study. Performance is defined in this context by the weight each model obtains in the BMA scheme. BMA weights represent model probabilities that reflect how well the model performs in reproducing the observed data, relative to the other models in the considered set. In that sense, they are a measure of the uncertainty about model selection, that is, the uncertainty about identifying the single true model in the set of candidate models (see Höge et al. 2019 for more details). BMA weights can therefore be used as a criterion for model selection (or rejection).

BMS is an objective way to evaluate the performance of alternative model structures that comprise a pool of possible descriptions of the natural system. Ideally, the ensemble of alternative models would cover the entire “model space,” that is, all possible alternative models, and also contain the “true model,” the model that has generated the data. In real‐world applications, however, the true model does not exist and the number of models to use is limited—mainly for practical reasons such as the effort to set up the models. Methods to sample the model space more widely have emerged in hydrology (Clark et al. 2008; Fenicia et al. 2011; Spieler et al. 2020) but they are still limited in their applicability, and no such framework exists for different model categories and for groundwater level simulations.

The BMA scheme used here was adapted from the work presented in Schöniger et al. (2015); Wöhling et al. (2015) and the earlier work by Hoeting et al. (1999). The set of models considered is defined as $M_{k}$, *k* = 1…*N*_*m*_, where *N*_*m*_ = 6 is the number of models. Each model defines a functional relationship *f*_*k*_ between its parameters ***u***_*k*_ and its predictions ***y***_*k*_ which corresponds to a vector of observations ***y***_*o*_ of length *N*_*s*_:

(1) $$M_{k}:y_{k}=f_{k}\left(u_{k}\right).$$

The posterior predictive distribution of the quantity of interest Δ (here: groundwater levels) given ***y***_*o*_ is determined as a weighted average of the individual models' predictive distributions:

(2) $$p\left(\Delta |y_{o}\right)=\sum_{k=1}^{N_{m}}p\left(∆|y_{o},M_{k}\right)P\left(M_{k}|y_{o}\right).$$

The posterior weight for each model, $P\left(M_{k}|y_{o}\right)$, is determined using Bayes' theorem by:

(3) $$P\left(M_{k}|y_{o}\right)\propto p\left(y_{o}|M_{k}\right)P\left(M_{k}\right),$$

with prior model probabilities $P(M_{k})$ (here set to 1/*N*_*m*_, i.e., all models weighted equally). The model weights determined by Equation 3 are then normalized by the sum over all weights to ensure they add up to unity:

(4) $$P\left(M_{k}|y_{o}\right)=\frac{p\left(y_{o}|M_{k}\right)P\left(M_{k}\right)}{\sum_{j=1}^{N_{m}}p\left(y_{o}|M_{j}\right)P\left(M_{j}\right)},$$

where $p\left(y_{o}|M_{k}\right)$ is the average likelihood that an individual model has generated the data set ***y***_*o*_ (also known as the Bayesian model evidence, e.g., Schöniger et al. 2014):

(5) $$p\left(y_{o}|M_{k}\right)=\int p\left(y_{o}|M_{k},u_{k}\right)p\left(u_{k}|M_{k}\right)du_{k}.$$

The data likelihood function in Equation 5 is here assumed to be Gaussian with the mean equal to the prediction ***y***_*k*_ and an error covariance matrix ***R***:

(6) $$\begin{array}{ll} p\left(y_{o}|M_{k},u_{k}\right) & =2\pi^{-N_{s}/2}|R{|}^{-1/2}\\ & \;\exp\left[-\frac{1}{2}{\left(y_{o}-y_{k}\right)}^{T}R^{-1}\left(y_{o}-y_{k}\right)\right], \end{array}$$

where ***R*** is a diagonal matrix of size *N*_*S*_ × *N*_*S*_. In view of their respective value ranges, we chose a measurement error standard deviation of 5.5/4.5/3.0/3.0 m for the monitoring wells P3010/P3821/P3954 and P3009, respectively.

It should be noted that all resulting probability functions and statistics are implicitly conditional on the set of considered models *M*_*k*_ and the information content of the observations ***y***_*o*_. For further information on the BMA scheme, the reader is referred to Wöhling et al. (2015).

A priori, each model has a weight of 1/*N*_*m*_ = 0.167. The posterior model weights (BMA weights, Equation 4) can range between 0 and 1. A model obtains a weight of 1, if it has generated the data and/or if no alternative models exist in the ensemble with a weight >0. For the interpretation of BMA weights, however, it should be noted that a weight of 1 is not necessarily an indication that the model fits the data perfectly. Instead, it indicates that it is the best model in the model set by “some distance” compared to the alternative models. Similarly, a BMA weight of zero does not necessarily mean the model represents the data poorly. It implies that at least one other candidate model performs “better” and no additional information is provided by the model with zero weight. BMA weights are a means for ranking models in light of existing data and therefore, care should be taken when discarding models based on low model weights. The BMA weights and model ranking might be different for other, yet unknown, data.

The BMA/S framework is generic and can accommodate any type of multi‐model ensemble. It was used here to rank models in a diverse ensemble with models of different types, largely different numbers of model realizations (Table 1) and also different ways the realizations were generated as described above. The existing ensemble can easily be evaluated against new members and new model types. It can also be extended with realizations that represent the uncertainty of model inputs in addition to parametric uncertainty. In contrast to most BMA applications, we did not use unconditional model realizations (i.e., model realizations sampled from a prior distribution of model parameters) in our BMA scheme. Instead, the priors were (distributions of) model realizations that have been calibrated on data. Models were treated as predictors that compete against each other independently of their type or method of construction according to a uniform standard that was only set by the data (Wong and Clarke 2019). As a result of BMA, we obtained a multi‐model distribution that envelopes all forecast scenarios that seem plausible given the current data (Höge et al. 2019).

## Results and Discussion

The results of this study are presented in two parts. First, the performance of the individual models was evaluated by selecting the individual best‐fit model realizations for each model and monitoring well. In the second part, the multi‐model ensembles of the monitoring wells were analyzed by BMA methods.

### Individual Model Evaluation

**Figure 2:**
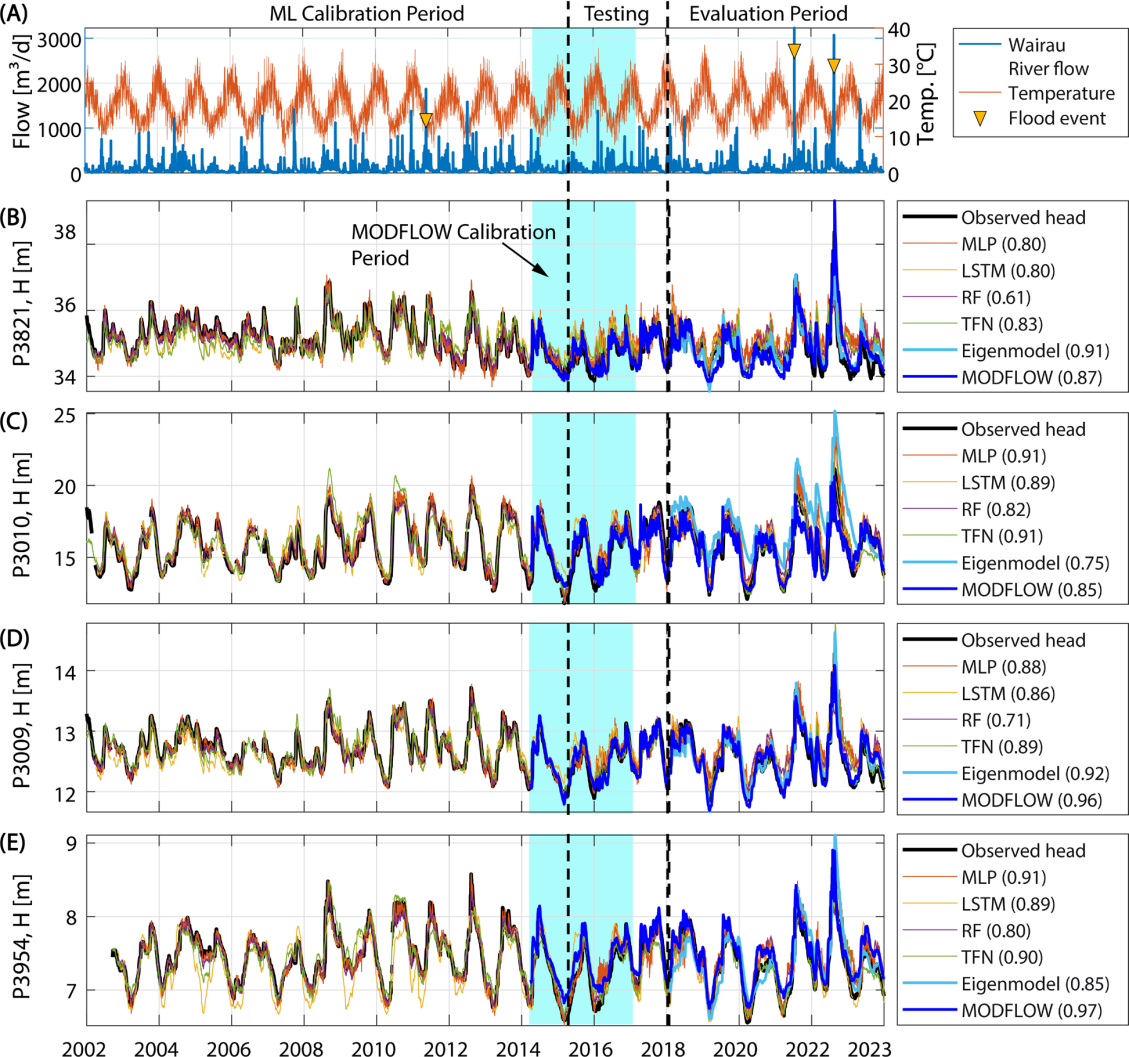
Summary plot of observed and simulated groundwater levels for wells P3821 (B), P3010 (C), P3009 (D) and P3954 (E) and the individual models using their best‐fit parametrizations with respect to the KGE criterion (for the evaluation period). Wairau River flows and air temperature are shown in panel (A). Vertical dashed lines and the shaded blue area indicate the calibration, testing and evaluation periods.

The results of the model comparison are summarized in Figure 2 and Tables 2 and 3 and subsequently described. First, we selected from each model ensemble the one realization with the largest KGE value in the evaluation period. The simulated groundwater levels corresponding to these best‐KGE‐fit realizations were plotted in Figure 2 for the four monitoring wells and for both the calibration and evaluation periods. The groundwater levels were plotted in units of meter above sea level and ordered from west to east (high to low) following the general groundwater flow direction toward the coast (Figure 1).

**Table 2:** Performance of the Models for the Individual Best‐Fit (Based on KGE) Parameter Sets and the Model Evaluation Period

Model	RMSE	*R* ^2^	Bias	KGE	Rank
**P3821** MLP	0.47	0.70	−0.43	0.80	4
LSTM	0.42	0.77	−0.38	0.80	4
RF	0.38	0.62	−0.28	0.61	6
TFN	0.31	0.76	−0.27	0.83	3
EM	**0.23**	**0.83**	**−0.12**	**0.91**	1
MOD	1.38	0.77	−1.38	0.87	2
**P3010** MLP	0.58	0.85	−0.29	**0.91**	1
LSTM	0.50	**0.90**	−0.16	0.89	3
RF	**0.47**	**0.90**	**−0.07**	0.82	5
TFN	0.69	0.83	0.39	**0.91**	1
EM	1.35	0.64	−1.15	0.75	6
MOD	0.64	0.81	−0.31	0.85	4
**P3009** MLP	0.21	0.82	−0.18	0.88	4
LSTM	0.20	0.86	−0.17	0.86	5
RF	0.20	0.80	−0.15	0.71	6
TFN	0.12	0.82	−0.04	0.89	3
EM	0.13	0.86	0.06	0.92	2
MOD	**0.10**	**0.91**	**−0.02**	**0.96**	1
**P3954** MLP	0.16	0.84	−0.12	0.91	2
LSTM	0.15	0.81	−0.02	0.89	4
RF	0.15	0.86	−0.10	0.80	6
TFN	**0.12**	0.81	**0.01**	0.90	3
EM	0.18	0.73	0.05	0.85	5
MOD	0.15	**0.95**	−0.14	**0.97**	1

**Table 3:** Performance of the Models for the Individual Best‐Fit (Based on RMSE) Parameter Sets and the Model Evaluation Period

Model	RMSE	*R* ^2^	Bias	KGE	Rank
**P3821** MLP	0.40	0.63	−0.32	0.66	6
LSTM	0.36	0.75	−0.30	0.66	4
RF	0.38	0.63	−0.28	0.60	5
TFN	0.24	0.72	**0.01**	0.79	2
EM	**0.21**	**0.83**	−0.03	**0.90**	1
MOD	0.29	0.73	**−0.002**	0.75	3
BMA mean	**0.21**	0.82	**−0.02**	0.90	—
**P3010** MLP	0.58	0.85	−0.29	**0.91**	3
LSTM	0.50	**0.90**	−0.16	0.89	2
RF	**0.46**	**0.90**	−0.07	0.82	1
TFN	0.69	0.83	0.39	**0.91**	5
EM	1.34	0.64	−1.13	0.75	6
MOD	0.59	0.83	**−0.02**	0.75	4
BMA mean	**0.46**	**0.91**	**−0.07**	0.82	—
**P3009** MLP	0.19	0.75	−0.12	0.83	5
LSTM	0.16	0.90	−0.12	0.81	4
RF	0.20	0.80	−0.15	0.71	6
TFN	0.12	0.82	−0.02	0.86	2
EM	0.13	0.86	−0.03	0.91	3
MOD	**0.10**	**0.91**	**0.01**	**0.95**	1
BMA mean	**0.11**	**0.92**	−0.07	0.91	—
**P3954** MLP	0.16	0.79	−0.08	0.85	5
LSTM	0.13	0.83	−0.04	0.86	3
RF	0.15	0.86	−0.10	0.79	4
TFN	0.12	0.81	**0.01**	**0.90**	1
EM	0.17	0.74	−0.04	0.84	6
MOD	**0.08**	**0.95**	**0.01**	0.89	2
BMA mean	**0.09**	**0.95**	−0.06	0.87	—

Note: The performance of the ensemble (BMA) mean is also shown.

The range of groundwater level changes is different for each of the individual wells as can be noted by the different scale of the y‐axes in Figure 2. For example, the range of groundwater level fluctuations for well P3010 is more than 12 m, whereas it is only slightly more than 2 m for well P3954. It should also be noted that the evaluation period contains two flood events in the Wairau River (on July 18, 2021 and August 21, 2022) which were among the largest floods on record and by far exceeded the largest flood (on May 26, 2011) in the calibration (and testing) period. This makes our model evaluation more robust because it contains system states not previously seen by the model. Particularly, the data‐driven ML techniques have been frequently criticized for not being able to predict outside of their calibration data range (e.g., Murphy 2012). This could be tested in our evaluation framework.

#### Performance of the Best‐Fit Models Based on the KGE Criterion

The performance of the KGE best‐fit models for the individual wells and the evaluation period are summarized in Table 2. The KGE values for all wells and models range between 0.61 and 0.97. For each of the wells, there is at least one model performing with KGE ≧ 0.90 (indicated in bold font in Table 2). However, it is not consistently the same model that fits the data best. For well P3854 and P3009, which are in the spring‐belt region, MODFLOW obtained the highest KGE values. For well P3010, which is furthest away from the river, the TFN and the MLP models perform equally well. EM performs best for well P3821. There are several models for all the wells that have only a slightly lower KGE value than the best one and fall in the category “good” (KGE ≧ 0.75). MODFLOW obtained consistently high KGE‐values in the model ensembles for all wells (Table 1) with an average KGE‐value of 0.91. The TFN, MLP, LSTM, and EM models obtained only slightly lower average KGE‐values ranging between $\bar{\mathrm{KGE}}=0.86$ and 0.88. The lowest model fit to the data was observed for the RF model ($\bar{\mathrm{KGE}}=0.74$). This indicates that, judged by the KGE criterion, most of the models are very similar in their performance. By the categorization defined above, these models perform “good”, whereas the RF model is performing “intermediate”.

Some other observations with respect to the performance of the best‐KGE models can be made. Most notably, for the wells P3010 and P3954, the best models do not rank best with respect to bias and correlation despite the fact that the KGE is a composite of these criteria (plus variance). This highlights the different skill score of different fitness criteria and underlines earlier findings that a single criterion may favor particular features in the model output and not necessarily lead to an “overall‐best” fit to all aspects of the data (e.g., Wöhling et al. 2013).

#### Performance of the Best‐Fit Models Based on the RMSE Criterion

In addition to the KGE‐best fit models described in the previous section, we also selected the models attaining the best (lowest) RMSE value from the model ensembles. The results are summarized in Table 3. The performance of the best‐RMSE models for wells P3821, P3009, and P3934 was 0.21, 0.10, and 0.08 m, respectively. The performance for well P3010 was lower, as indicated by an RMSE value of 0.46 m. MODFLOW performed best for wells P3009 and P3954, whereas the EM and RF models performed best for wells P3821 and P3010, respectively. The RMSE‐best model was also the KGE‐best model except for well P3010.

Similarly to what was observed for KGE above, the models selected based on RMSE don't always perform best in all other performance criteria (here, well P3009 is the exception, see Table 3). The model with the lowest average RMSE value across all four wells is MODFLOW with $\bar{\text{RMSE}}=0.27$, closely followed by LSTM and TFN ($\bar{\text{RMSE}}=0.29$) and RF ($\bar{\text{RMSE}}=0.30$). MLP and EM obtained higher average RMSE values (0.33 and 0.46, respectively), mainly because of their poor performance to well P3010. In general, well P3010 exhibits much larger model errors across all models compared to the other wells, which is likely related to its position at the southern margins of the Wairau Aquifer, with a more complex hydrogeological setting and some influence from ephemeral streams in the southern valleys of the Wairau Plain. As a result, the seasonal variation of groundwater levels is relatively large in this well compared to the other wells and can exceed 5 m (Figure 2C). The EM seems to suffer most from structural errors and exhibits the by far largest errors.

#### Extrapolation Skill

**Figure 3:**
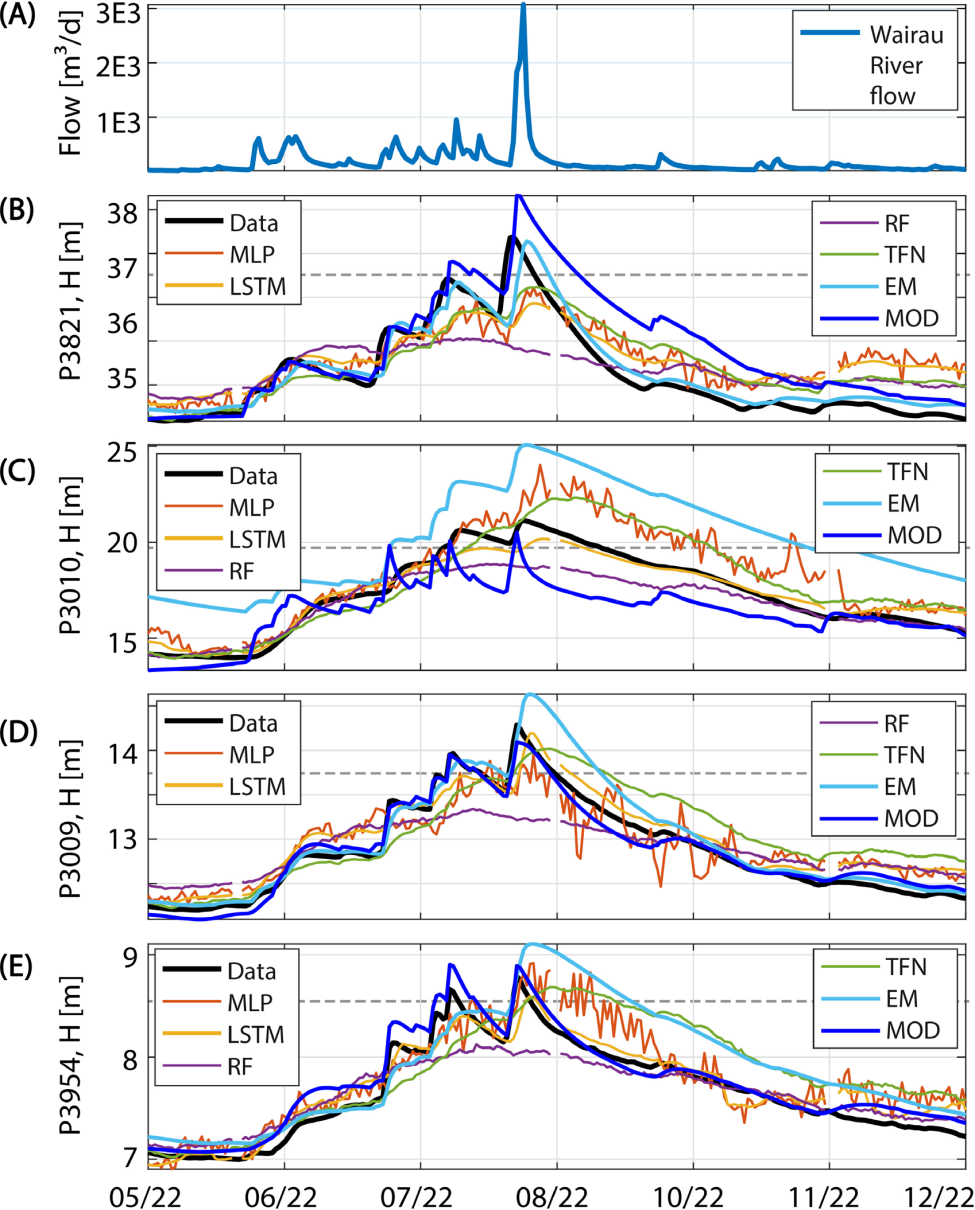
Observed and simulated groundwater levels during the August 2022 flood event for the wells P3821 (B), P3010 (C), P3009 (D) and P3954 (E). Model simulations with the RMSE‐best‐fit parametrizations are shown. Wairau River flow is depicted in panel (A). The dashed lines represent the maximum groundwater levels in the calibration period.

The evaluation period contains two major flood events that represent extremes not observed in the calibration/training period. While the differences between models appear to be generally relatively small for the 21‐year calibration and evaluation period (Figure 2), more variability among the models is visible during these flood events. This is exemplarily discussed for the extreme flood event in August 2022. The corresponding model simulations for the four wells and the individual models are shown in Figure 3.

Groundwater levels were increasing in all wells from June 2022 onward to the peak of the event on August 21 due to extended periods with river flow well above the mean of 100 m^3^/s. The 2022 flood was a very flashy event with a sharp increase to the peak and a steep recession afterwards. The groundwater response was strongly attenuated, but resulted in the largest groundwater levels observed in the considered time period of the study in all of the wells. This behavior was reproduced by the models with varying degrees of accuracy. MODFLOW mimics the groundwater level dynamics and also exhibits a good fit to the flood peak for wells P3009 and P3954. But it overestimates/underestimates the peak in P3821 and P3010, respectively. The EM overestimates the peak levels for all wells except P3821. The ML models exhibit mixed results. RF predictions are lacking the dynamics of the observations, and MLP simulations are generally very noisy. The latter may be an indication of overfitting, but was also observed for settings with lower degrees of freedom (results not shown). The best performing ML model for the flood event across all wells is the LSTM, which reproduces the dynamics well and also fits the peak values sufficiently in three out of the four wells (Figure 3).

Interestingly, the LSTM did not rank first for any of the wells based on the KGE or RMSE value, but exhibits a consistently good performance for all wells (see above). These results illustrate that different models have different strengths and weaknesses, and they may fit the data at particular times better than at others. It also shows that model selection is dependent on the question of interest, and models should not readily be discarded from a model ensemble simply because another model appears to be more accurate judged by a single selection criterion. This topic will be discussed in more detail later.

Another aspect that was analyzed on the extreme flood event is the model's ability to predict groundwater levels that lie outside of the range of their calibration data. The maximum groundwater levels of the training/calibration period are indicated by horizontal dashed lines in Figure 3B through 3E. If model predictions lie above the threshold, the model is able to extrapolate beyond the calibration data. This is clearly the case for the EM model, but also for the MOD, LSTM, TFN, and MLP models. RF seems not to be able to extrapolate beyond the calibration range in our application.

#### Considerations for Practical Application of the Individual Models

The models analyzed in this study are members of different model classes, ranging from physics‐based (MOD), conceptual (EM, TFN) to ML models (RF, LSTM, MLP). Each model has its strengths and weaknesses and it largely depends on the model purpose and the (sometimes subjective) preference of the modeler which model ranks best. The characteristics and dynamics of the groundwater observations to which the models are applied are likely to determine the model ranking. There is no “model that fits all” and thus, the individual model's performance and the resulting model choice may be often specific to the site under investigation. However, there are some general considerations and observations that can be made based on the results reported above.

As a starter, there is a significant difference in both the motivation and purpose of using spatially explicit models based on PDEs like MODFLOW (or, e.g., ParFlow, HydrGeoSphere, FEFLOW, COMSOL) and data‐driven (ML) methods in particular. A transient MODFLOW simulation yields a time series of groundwater level fields for the entire model domain, not only at a single location. The simulation results for individual monitoring wells belong to the same model solution (and parameter set) and thus are conditional on each other and often also conditional on other calibration targets. In contrast, individual models are constructed for each well separately for the conceptual and data‐driven models in this study, and no such interdependency of model parameters or targets exists.

This is a major difference between MODFLOW and all other models and—admitting inevitable model deficiencies—intuitively one could expect that it is more difficult to fit all four wells at once compared to each well individually. However, this also depends on the degrees of freedom (or target‐sensitive parameters) each individual model has to fit the data. This is not easy to compare between different model types. While MODFLOW parameters usually have a physical expression in the real world, parameters in ML models do not have this relationship. ML models often require the specification of only a few (hyper)parameters but some of these (e.g., the number of nodes and layers in MLP or the number of trees in RF) can result in highly complex model architectures with many parameters to adjust in the model calibration phase. Thus, ML models are not necessarily less complex than PDE‐based models, but they are simpler to set up.

Another difference between the model types is their purpose. PDE‐based models are used to integrate different data (types & locations), are at least to some degree bounded by the laws of physics, which allows for better interpretability and system understanding but also for model diagnostics and hypothesis testing. This usually comes along with a higher parametrization effort and thus with a high(er) need for data and computational resources. For example, the MODFLOW model in this study has 572 parameters and requires approximately 1 h for a single run (10 years) on a standard 8th generation Intel™ Core®i7 CPU. The input data requirements are very high as the relevant meteorological model forcings need to be defined in high resolution in space and time (Table 1). In addition, the model setup and calibration of MODFLOW require substantial resources and skills, can take days to weeks, and is typically not a sequential but rather an iterative process. This is the reason why the calibration period for MODFLOW is significantly shorter than for any other model (Figure 2).

In contrast, the EM requires substantially less data, fewer parameters (10 in the setup used here) and a single model run takes less than 0.01 s on the CPU described above. Instead of spatially distributed inputs, meteorological data from a single station is sufficient (Table 1). The same applies to TFN, RF, MLP, and LSTM. These models are also very fast (run times <1 s) which allows their application in operational forecasting and ensemble modeling as demonstrated by the AquiferWatch tool with the EM model (Wöhling and Burbery 2020). In addition, the effort to set up and calibrate/train these models is much lower; it can typically be done within the time span of a day or less. The effort scales with the number of wells in the aquifer (as one model for each well has to be setup, calibrated and executed) but the task can easily be parallelized. However, the data requirements and run times are still much lower than for MODFLOW. Therefore, if the model's purpose is solely the simulation of groundwater levels, MODFLOW (type) models may not be the best choice. PDE‐based models have been advocated for their ability to extrapolate beyond the range of historic data, but our results suggest that ML and conceptual models are also able to extrapolate beyond the training range. In addition, the physical interpretability of these models is limited, and there is no guarantee of a comparable performance under different (combinations of) extreme model forcings.

### Ensemble Modeling

The BMA/S framework adopted here allows us to analyze predictive distributions of model results as opposed to identifying each model's single best‐fit solution that was described in the previous sections. The framework also offers an arguably less subjective method for model ranking and selection compared to using best‐KGE/RMSE realizations since the entire predictive distribution is evaluated and all assumptions about the prior belief in individual models, individual data types and values, as well as errors are made transparent in the scheme. In this section, we present first the predictive uncertainty of the individual models and then we discuss the results of the BMA/S scheme.

#### Predictive Uncertainty of Individual Models

**Figure 4:**
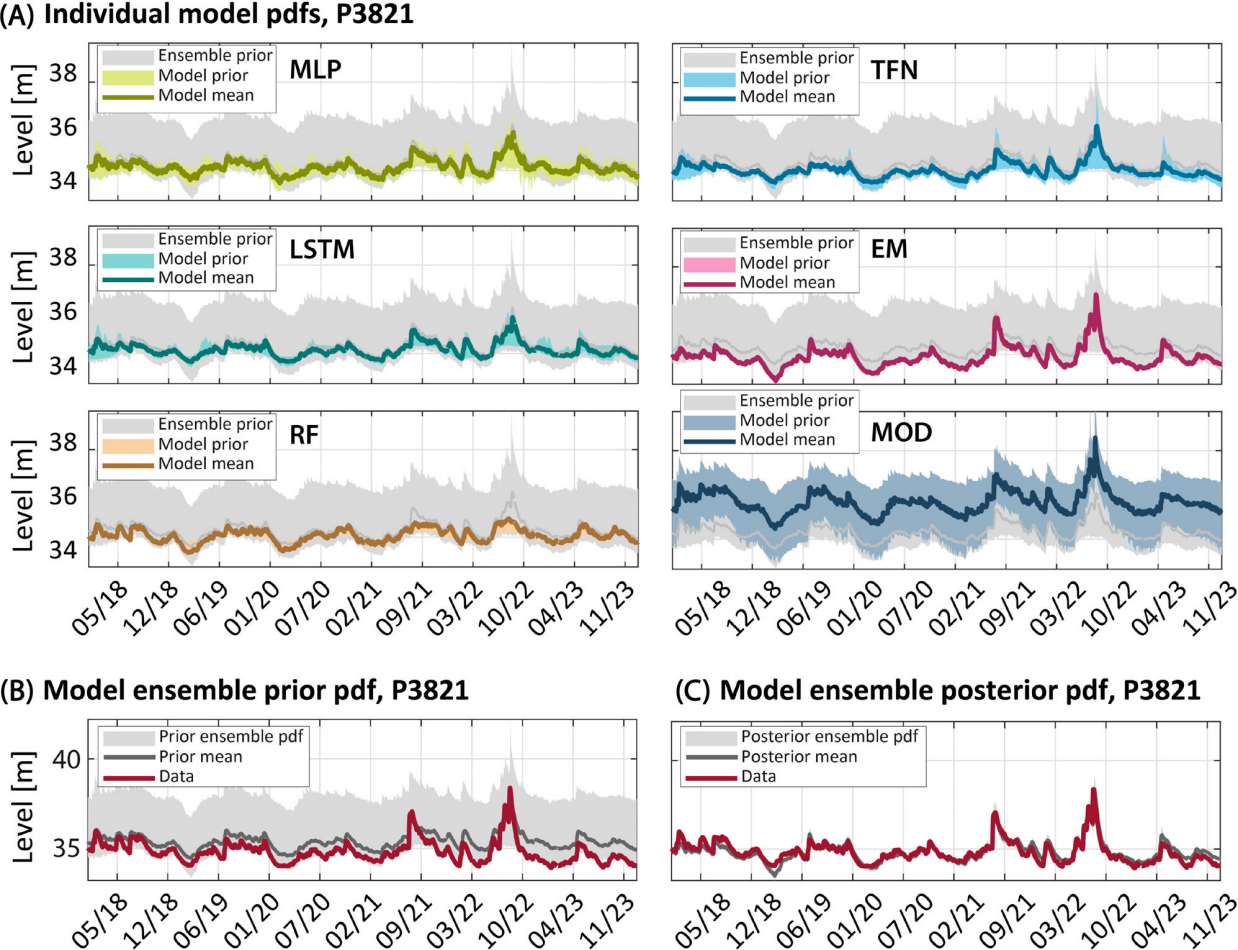
Ensemble simulations for well **P3821**: (A) 95% uncertainty bounds of the individual model distributions (colored area) and model means for MLP, LSTM, RF, TFN, EM, and MOD. The 95% uncertainty bounds of and the mean of the prior model ensemble are shown in (B) and additionally in all panels of (A). Uncertainty bounds of the posterior ensemble and the BMA mean are depicted in (C).

**Figure A1:**
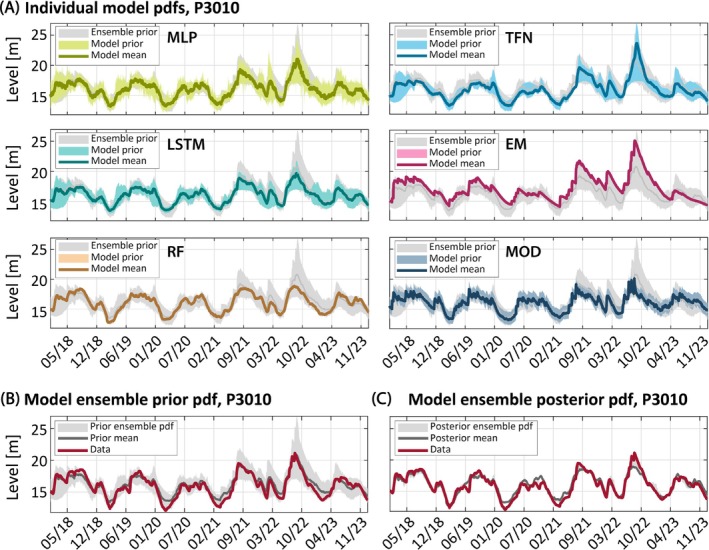
Ensemble simulations for well **P3010**: (A) 95% uncertainty bounds of the individual model distributions (colored area) and model means for MLP, LSTM, RF, TFN, EM, and MOD. The 95% uncertainty bounds of and the mean of the prior model ensemble are shown in (B) and additionally in all panels of (A). Uncertainty bounds of the posterior ensemble and the BMA mean are depicted in (C).

**Figure A2:**
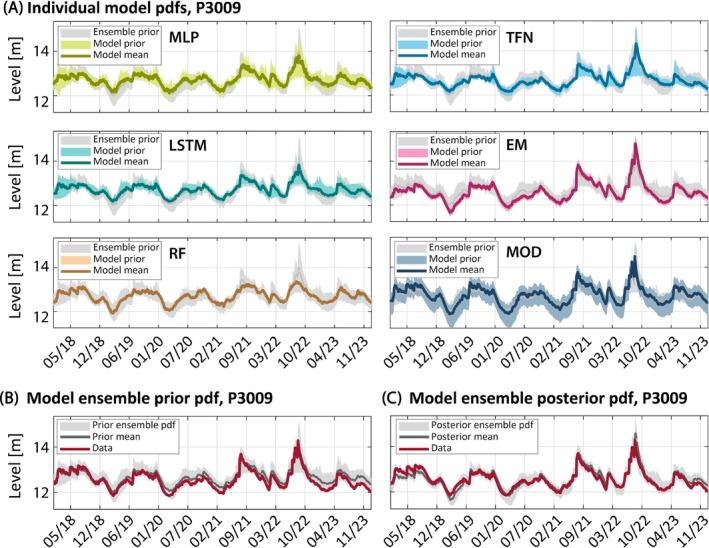
Ensemble simulations for well **P3009**: (A) 95% uncertainty bounds of the individual model distributions (colored area) and model means for MLP, LSTM, RF, TFN, EM, and MOD. The 95% uncertainty bounds of and the mean of the prior model ensemble are shown in (B) and additionally in all panels of A). Uncertainty bounds of the posterior ensemble and the BMA mean are depicted in C).

**Figure A3:**
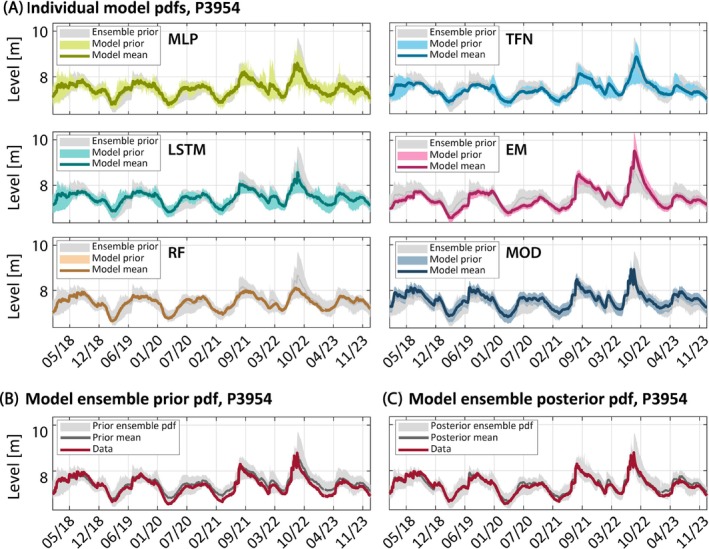
Ensemble simulations for well **P3954**: (A) 95% uncertainty bounds of the individual model distributions (colored area) and model means for MLP, LSTM, RF, TFN, EM, and MOD. The 95% uncertainty bounds of and the mean of the prior model ensemble are shown in (B) and additionally in all panels of (A). Uncertainty bounds of the posterior ensemble and the BMA mean are depicted in (C).

Uncertainty ranges of the individual models for groundwater level predictions for well P3821 and the evaluation period (January 2018 to December 2023) are exemplarily depicted in Figure 4A. The corresponding results for the wells P3010, P3009, and P3954 are shown in the Appendix (Figures A1A to A3A), respectively.

The 95% prior uncertainty ranges and the model mean of the individual models are shown by a colored shaded area and a colored solid line, respectively. Since the prior model bounds stem from calibrated (distributions of) model realizations, most of the models exhibit relatively small uncertainty bounds. Only MODFLOW exhibits a wider uncertainty range (Figure 4A) which is likely the result of equifinality of the highly‐parametrized model paired with the shorter calibration period length.

The uncertainty ranges of the EM and RF models are very small and hardly visible in the plot, which is likely caused by over‐confidence or over‐fitting (or both) in the calibration process in combination with the low number of ensemble members in the case of RF. These patterns are consistent and can be found for all other wells too (see Figures A1A to A3A).

#### Ensemble Predictive Uncertainty

Individual BMA runs were performed for each of the four monitoring wells and the evaluation period using the multi‐model ensembles that consist of the post‐calibration ensembles of the six individual models. Figure 4B shows exemplarily for well P3821 the 95% prior uncertainty ranges and the model mean of the multi‐model ensemble by the gray shaded area and a darker gray line, respectively. The same shades are also included in the individual panels of Figure 4A such that the colored area depicts the predictive uncertainty of the individual model, while the gray area shows the predictive uncertainty of all models combined in the multi‐model ensemble.

As expected, in all cases tested in this study, the individual models' uncertainty range is smaller than the uncertainty range of the prior ensemble. The multi‐model prior uncertainty range for well P3821 is dominated by the relatively large uncertainty range of the MODFLOW model, but this is not the case for all other wells. In addition, MODFLOW predicts on average higher groundwater levels than all the other models which is likely a reflection of the less constrained west–east gradient of groundwater levels on the upper Wairau Plain and potentially some influence of the generally steeper groundwater level gradients from the southern Hills. Most observations for well P3821 are still encapsulated by the uncertainty bounds of MODFLOW but the results demonstrate that in case of larger structural uncertainty there is some benefit of building model ensembles with locally predicting models. However, spatially explicit modeling with MODFLOW can also give predictions at arbitray locations where no data is available and where local models are not applicable.

The prior ensemble mean is already a good predictor for well P3821 since most models exhibit a good fit to the data (Figure 4A and 4B). The posterior ensemble (BMA) mean is depicted in Figure 4C. The performance is similar to the one of the best individual model with a slightly lower bias but also slightly lower *R* ^2^ value than the EM model (Table 3). The posterior uncertainty ranges are much smaller than the priors, arguably too small and hardly visible in the graph. This is a reflection that most models agree with each other and that most MOD realizations fall outside the 95% posterior uncertainty bounds. Another factor for these over‐confident model bounds are the assumptions related to the error structure in the BMA/BS scheme. The model residuals are not independent and normally distributed (an example is shown in the Supporting Information). One option would be to relax these assumptions and replace the likelihood function (Equation 6) with a different distribution and/or extend it by an (auto)correlation function. This has been adopted in Bayesian model inference before, for example, by Schoups and Vrugt (2010); Woodward et al. (2017) and others, but the application is not always successful (see discussion in Wöhling and Vrugt 2011).

The results for the wells P3010, P3009, and P3954 are very similar to the results discussed above, but there are also a few differences. As shown in Figure A1B, the prior ensemble mean for well P3010 exhibits a larger mismatch to the data despite the fact that the prior ensemble consists of models that are already conditioned on data (although on a different time period). The performance of the BMA mean, however, is again comparable (or slightly better) to the best model (RF) in the ensemble (Table 3). The posterior uncertainty bounds are again very small (Figure A1C), but this is not the case for the wells P3009 and P3954, where the uncertainty bounds are wider and cover the data much better (Figures A2C and A3C).

In general, we would like to make the argument that prediction with a diverse model ensemble is more robust in terms of predictive power than predictions with a single model. BMA is a suitable and transparent framework for model ranking, averaging and selection. The rankings and weights are always conditional on the data but BMA weights can easily be updated as new data becomes available. Further, the BMA framework can also help to make informed choices to choose from a set of existing models and construct model ensembles that are both accurate and efficient in terms of computational and data requirements.

#### Bayesian Model Selection and Posterior Model Weights

**Figure 5:**
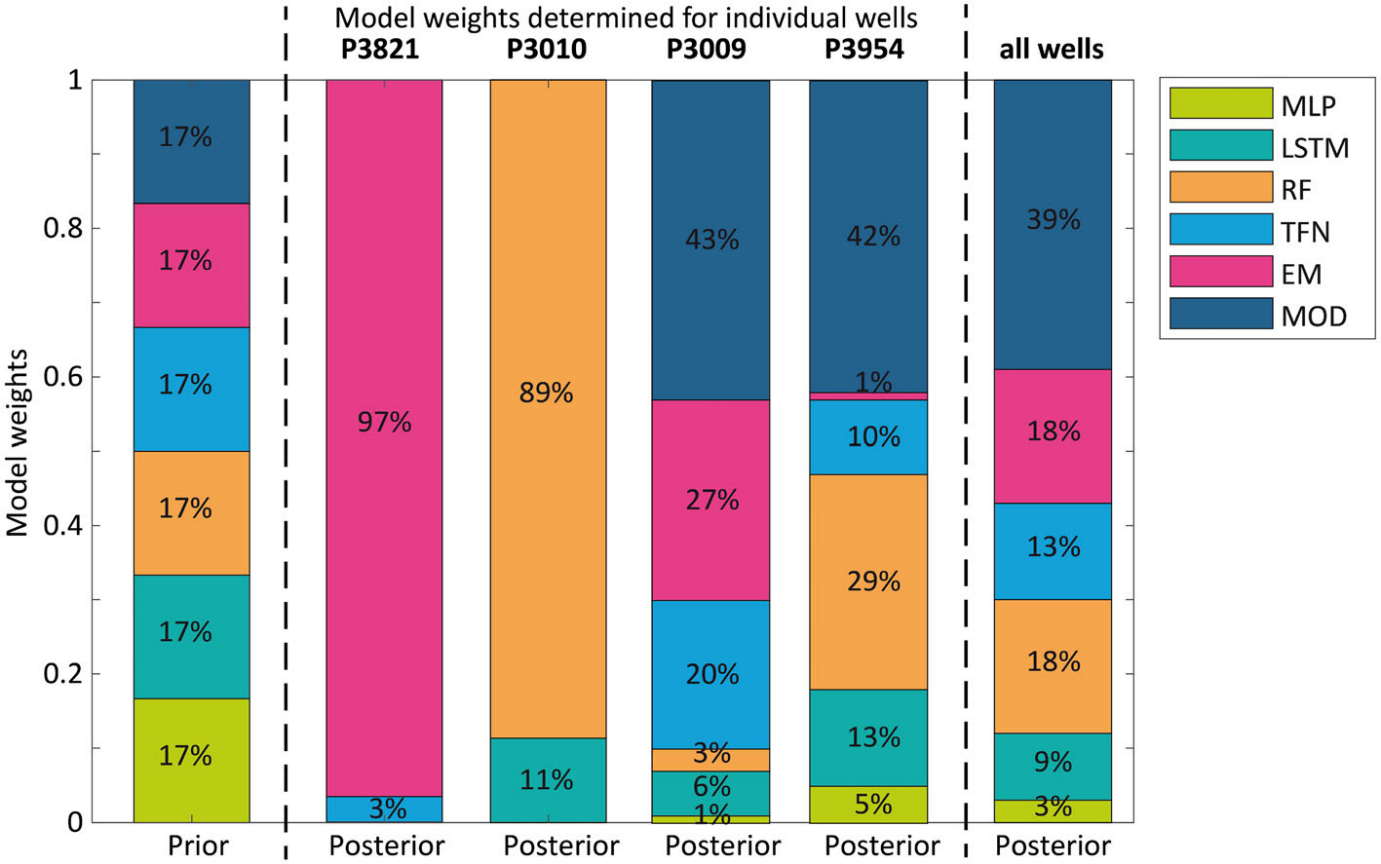
BMA model weights obtained for the evaluation period.

An outcome of the individual BMA runs is posterior model weights (Equation 4) derived for the evaluation period. The posterior model weights are depicted in Figure 5 together with the corresponding prior model weights. The weights are expressed as percentages (and sum up to 100%) and models not explicitly visible in the bar diagram have obtained a weight of zero.

When ranking models by their BMA weight, it can be seen that the EM model ranks first (obtained the largest weight) for well P3821 (97%), the RF model ranks first for well P3010 (89%) and MOD ranks first for wells P3009 and P3954 (43% and 42%). EM and RF both dominate the ensemble only in one of the wells and obtain weights close to 30% in one other well (P3009 and P3954, respectively). For the other wells, significant contributions come also from other models (Figure 5). The BMA runs for wells P3009 and P3954 resulted in a wider spread of weights among the models in the ensemble, but MLP received consistently low weights.

The model ranking based on BMA weights shows some similarities but notable differences to the rankings based on best‐KGE and best‐RMSE solutions. As discussed above, the RMSE has similarities to the Gaussian likelihood used in the BMA/S scheme, but is not weighted by the measurement error. The best RMSE ranking models only obtained the largest posterior BMA weights in three of the four wells, and there are also differences in the lower ranks for wells P3009 and P3954 (Table 3). While TFN obtained the best (lowest) RMSE values for well P3954, it got only 10% of the posterior weights in the BMA scheme. The differences in BMA and KGE model rankings are even larger; only for well P3821 is there agreement that the EM ranks first (Table 2 and Figure 5).

There is no clear pattern of BMA weights for the four wells in this study. Although MODFLOW obtained the largest weights in two of the four wells, there are also significant contributions from other models in their multi‐model posterior distributions. No single model dominates or outperforms the others when the individual wells are investigated independently. Some models got a weight of zero although their best fit to the data is “good” according to the KGE criterion. For example, MLP, LSTM, and MOD get zero weights for well P3821 (Figure 5) but obtained KGE values of 0.80 or larger (Table 2). Another example is the TFN model for well P3010 with a KGE of 0.91. A possible explanation of these results is overfitting. This underlines the earlier statement that BMA weights are a measure for ranking model distributions but not necessarily a criterion for model accuracy.

To investigate the question of model choice and the (lack of) model preference for the four wells tested in this study, we performed another BMA run, where all four wells were simultaneously defined as prediction targets. The resulting model weights are shown by the right column in the bar plot of Figure 5. Now, all six models obtained weights greater than zero, but the ensemble is dominated by MODFLOW (39% of the weights). EM and RF posterior weights (18%) are close to their prior weights, and MLP ranked lowest at only 3%. This result illustrates the merits of MODFLOW as a “general” predictor because it competes in our BMA run with the set of locally calibrated models. Therefore, we would expect its performance to be similar or better also in comparison to global ML models that are currently emerging in the scientific literature (e.g., Iraola et al. 2024; Wetzel et al. 2024).

Our results demonstrate that there can be a degree of subjectivity in model choice, particularly when using “best‐fit” criteria for model choice. The BMA/S framework can mitigate this subjectivity, or at least make assumptions explicit. Since BMA weights are always conditional on the data set, they may change when new data becomes available and thus models with low BMA weights but acceptable data fits should not lightly be discarded from the model ensemble.

## Summary and Conclusions

The performance of six selected model classes to predict groundwater levels was tested on data from four wells of the Wairau Aquifer in New Zealand. The model set comprised a mechanistic model (MODFLOW), two conceptual models (EM, TFN) and three data‐driven ML models (MLP, LSTM, RF) with largely different requirements to set up, for input data and parameterization. We ranked the models by the performance of best‐fit solutions that were selected using two common model evaluation criteria, the KGE and the RMSE. Then we applied BMA to derive posterior model weights and predictive distributions. The weight‐based rankings were compared to the best‐fit ranking results, and the model ensemble predictions evaluated.

The main conclusions from our study can be summarized as follows:

- No single model exists in our model ensemble that outperforms all other models when tested separately on individual wells, regardless of the model ranking method.
- The choice of the model ranking criteria influences the ranking results: KGE and RMSE favor different models for some but not all wells. The ranking by BMA‐derived weights is different again, but BMA provides a more objective framework that is based on the modeler's confidence in the data. When tested on all wells simultaneously, MODFLOW obtains the largest model weights and dominates all other candidate models in the ensemble.
- The performance, data requirements, and run‐times of the ML models are competitive with those of the conceptual models and much lower than for MODFLOW. MLP and LSTM can predict beyond their calibration range, and particularly, LSTM performs well for extremes. RF is not capable of extrapolation with the chosen configuration and shows the lowest accuracy for extreme floods.
- The physics‐based model MODFLOW fares well in the model comparison despite the fact that it has been calibrated on fewer data and is forced to make predictions for all wells simultaneously while all other models were calibrated (and thus had the chance to fine‐tune) for each well independently. The physical process description by MODFLOW pays off in the simultaneous evaluation of all four wells and does not get outperformed by local fine‐tuning of the data‐driven and hybrid models.
- The effort to set up, parameterize, and calibrate MODFLOW, however, is huge compared to the effort for the other models. In addition, the computational requirement for a single MODFLOW run is up to 1000 times larger. Thus, ML models are an attractive alternative when used for (ensemble) forecasting.
- Our findings suggest that for individual, local prediction targets, optimized prediction skill might be achieved by using several cutting‐edge ML methods and identifying the best‐performing one. If interested in predictions at several locations within a spatially connected system, a spatially integrated PDE‐based model still seems to pay off, if the effort in model building is justifiable (see also discussion of defensible model complexity in Guthke 2017). Nevertheless, the setup of several competing ML approaches also presents a substantial effort, so modeling objectives should be carefully checked beforehand.
- BMA/BS provides an objective framework for ensemble modeling and helps make informed decisions for model selection to generate computationally efficient model ensembles from a set of candidate models.

These findings open up several opportunities for future research. This includes the processing of more complex ensembles of candidate models, potentially generated by automated model structure generators and the inclusion of model realizations that represent not only parametric and model structural uncertainties, but also the uncertainty in model inputs.

## Authors' Note

The authors do not have any conflicts of interest or financial disclosures to report

## Supporting information


**Data S1** Detailed model description, model evaluation criteria and model residuals.

## Data Availability

The data that support the findings of this study are openly available in Zenodo at https://zenodo.org/records/14845858.
