# Supplementary material for: Comparing Physics‐Based, Conceptual and Machine‐Learning Models to Predict Groundwater Levels by BMA
Source: Ground Water. 2025 Apr 21;63(4):484–505. doi: 10.1111/gwat.13487 (PMC12272009; doi:10.1111/gwat.13487)
Supplement: Supplementary file 1 — Data S1 Detailed model description, model evaluation criteria and model residuals. [file GWAT-63-484-s001.pdf]

Supporting information for: Comparing physics-based, conceptual and machine-learning models to predict groundwater levels by BMA

Thomas Wöhling[1,2], Alvaro Oliver Crespo Delgadillo[1], Moritz Kraft[1], Anneli Guthke[3]

1: Dresden University of Technology (TUD), Chair of Hydrology, 01069 Dresden, Germany.

2: Lincoln Agritech Ltd., Ruakura Research Centre, Hamilton 3240, New Zealand.

3: University of Stuttgart, Stuttgart Center for Simulation Science (SC SimTech), 70569 Stuttgart, Germany.

MODFLOW model (MOD)

As described in the MODFLOW model section of the main manuscript, the 3D groundwater flow model of the Wairau Aquifer was implemented in MODFLOW-NWT (Niswonger et al., 2011) and is a progression from the model described in Wöhling et al. (2018). The extended model domain ranges from the confluence of the Waihopai River in the west to the coast of the Pacific Ocean in the east and is bounded by the Richmond range hills in the North (Figure 1). The southern boundary is formed by the base of a ridge that divides the Wairau Plains and the Awatere Valleys in the South. The surface area of the model domain (393 km^2) was discretized by a regular grid of 200 x 200 m cells. Vertically, the Wairau Aquifer domain was described by three unconfined layers of highly conductive Rapaura gravels with spatially variable thickness and hydraulic properties (Wilson, 2016). The base of the model and the southern margins are formed by the low-permeability Speargrass Formation (e.g., Brown, 1981). In the east, the unconfined Wairau Aquifer is increasingly confined by the overlaying Dillons Point Formation that extends to the coast and further offshore (Brown, 1981; Pondard and Barnes, 2010).

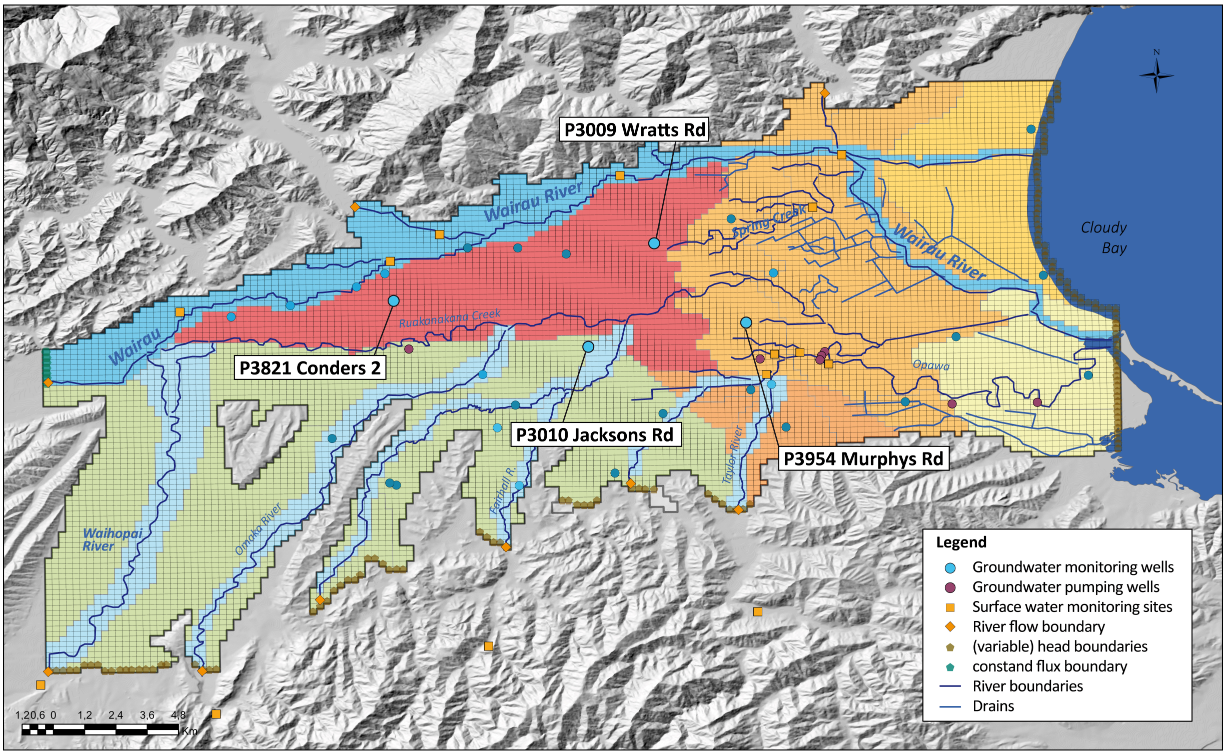

Figure 1: MODFLOW model domain and computational grid as well as the locations and type of boundary conditions and the location of monitoring wells used for calibration. The 4 monitoring wells used in this study are also shown. Coloured patches indicate hydrostratigraphic zones.

The different types of boundary conditions in the model, together with the chosen MODFLOW packages to implement them, are depicted in Figure 1 and listed as follows:

- 13 rivers, as well as their smaller tributaries, divided into 55 segments represented by the Stream-Flow Routing package (SFR, Niswonger and Prudic 2005). 9 of these rivers are provided with input time series, and three different types of diversions (priority, fraction and flood-control type) are used in the model.
- 6 networks of low-land springs or drainage canals modelled by the Drain package (DRN, Harbaugh 2005).
- Spatially distributed land-surface recharge and groundwater abstraction implemented by the Recharge and Well packages (RCH & WEL, Harbaugh 2005), respectively. Their inputs were computed externally by a spatially distributed soil-water balance model that has been described in detail by Wöhling et al. (2018). Furthermore, large groundwater abstraction wells for municipal and food

processing purposes were implemented as point sinks with metered daily pumping rates.

- Offshore groundwater flow / exchange at the sea was implemented with a variable head boundary condition in the constant-head package (CHD, Harbaugh 2005), representing a seasonal groundwater level derived from three coastal monitoring wells and extended all along the coastal boundary as a sinodial signal.
- Low-flow subsurface seepage as constant head boundaries (CHD package) along the entry points of the southern river valleys.
- Constant subsurface inflow along the Wairau River valley in the west via the flow and head boundary package (FHB, Harbaugh 2005).

A total number of 526 parameters of different types were used in model calibration (using PEST; Doherty, 2016a) to estimate unknown subsurface hydraulic characteristics and boundary flows:

- Hydrogeological parameterization is undertaken in 17 distinct hydrostratigraphical zones. Hydraulic conductivity is represented by 293 pilot points in 13 of these zones, while 4 low-conductivity zones were homogeneous with one parameter value each. Vertical anisotropy and specific storage were parameterized with 17 parameters, one for each zone, respectively, while specific yield parameters were distributed over 131 pilot points for the zones in the top layer of the model, and with 3 single parameters for the low-conductivity zones. Regularization has been applied to pilot point parameters of both hydraulic conductivity and specific yield using an experimental variogram. Thus, the spatial heterogeneity of the hydrogeology makes up the biggest part of the parameterization with a total of 465 model parameters.
- One parameter of hydraulic conductance was assigned to each of the six low-land spring and drainage networks.
- Similarily, each segment of the river network in the stream-flow routing package was assigned an individual hydraulic conductance parameter, leading to a total of 55.

We used the PEST software (Doherty, 2016a) and its utilities (ppk2fac, fac2real, int2real) for the parameterization setup with pilot point and zone parameters together with regularization.

For parameter calibration, a number of different observation types, as well as "soft" targets derived from expert knowledge were utilized. These observations are grouped into a single objective function to be minimized:

- 22 groundwater level time series from monitoring wells in different parts of the model region. Observations are weighted such that about 25% of the total weight goes to seven main monitoring wells in the Wairau Aquifer that have been identified as critical for the prediction of its overall water balance and availability of the low-land springs in the east, and another almost 24% to the remaining 15 groundwater level time series.
- A mixture of manual and automatically tracked measurements from seven different locations in five of the low-land springs and smaller streams in the eastern part of the model, with about 9% of the weights each assigned to time series of Spring Creek and Murphy's Creek, and about 7% of the weights to the remaining five locations.
- Two estimates of river-groundwater exchange based on several flow gauging campaigns in the Wairau River, implemented as observed river flow losses between gauging stations. Since the Wairau River is the main source of groundwater recharge, these observations have been given a weight of about 15% in the calibration scheme.
- Mean flow over the two constant / variable head boundaries in the southern (subsurface contribution of the southern river valleys) and eastern (offshore groundwater flow) parts of the model were estimated by experts from the Marlborough District Council and given a weight of 5% each.
- Several "binary" observations (yes / no) of flow in some smaller ephemeral rivers in the south-east of the model domain were given a weight of about 1% in the calibration.

The post-processing of model outputs to compare model simulations with these observations was conducted with the obs2obs tool in PEST.

Similar to the processes described in Wöhling et al. (2018); Wöhling (2019); Wöhling et al. (2020), subspace projection techniques were applied (Doherty, 2003, 2016b) for robust parameter estimation.

Eigenmodel (EM)

An eigenmodel is one of several methods to reduce the dimensionality of spatially-explicit 3D groundwater flow models that was first introduced by Sahuquillo (1983). Eigenmodels massively reduce the dimensionality of 3D flow models and are computationally cheap while maintaining some physical meaning of the considered groundwater system. Despite their promise, their use has been confined to a limited number of research groups (Bidwell, 2005; Bidwell and Burbery, 2011; Pulido-Velazquez et al., 2007a,b, 2008, 2011).

The eigenmodels used in this study have been set up previously by Wöhling and Burbery (2020) who developed the operational forecasting tool AquiferWatch for predicting groundwater storage under drought conditions. The eigenmodels consist of three main components: the actual eigenmodel approach to simulate the hydrodynamic response the aquifer system, a soil water balance model to compute land surface recharge and irrigation, and a river-recharge model. Land-surface recharge and irrigation abstraction were lumped over the domain and computed by the daily soil-water balance model adapted from Minhas et al. (1974) where actual evapotranspiration from the soil store is estimated by $ET_0(t)^{BRC}$ multiplied with a crop factor and a factor accounting for water stress. Soil water in excess of field capacity becomes groundwater recharge. River recharge is computed as a function of Wairau River flow using a function with a constant, linear, and exponential term (Wöhling and Burbery, 2020). The net recharge to the aquifer is then calculated as the sum of land-surface recharge and river recharge minus the groundwater abstraction for irrigation purposes, $Q_{abs}(t)$.

Eigenmodels represent the groundwater system as a set of linear reservoirs arranged

in series along a vertical slice of an aquifer with homogeneous and isotropic hydraulic properties. Drainage from each reservoir is proportional to the water in storage and controlled through a drainage coefficient that is the eigenvalue of the linear system. The first reservoir dominates the storage of the aquifer while successive reservoirs are important for describing the higher-frequency responses of the system. In the eigenmodels applied here, the number of reservoirs was chosen to be 5. Groundwater levels are computed by the eigenvalue solution to the transient groundwater flow problem (Sahuquillo, 1983) for perfectly connected streams (Pulido-Velazquez et al., 2005; Bidwell et al., 2008).

Post-calibration parameter ensembles for individual eigenmodels corresponding to the 4 monitoring wells were derived using Markov chain Monte Carlo (MCMC) simulation and the time period 01/01/2015 - 25/01/2018 as described in Wöhling and Burbery (2020). The calibration approach considers uncertainty in model parameters but also Wairau River flows. The four eigenmodel ensembles were then used for further analysis in this study. A summary of model inputs and parameters can be found in Table 1 of the main paper. For more details about the eigenmodel setup and calibration, please refer to Wöhling and Burbery (2020).

Transfer-Function Noise Model (TFN)

Transfer-Function Noise models relate input time series to output time series through a statistical model (von Asmuth et al., 2002). A common approach for the definition of this statistical model in the field of groundwater modelling has been the convolution of input time series with given impulse response functions to generate the output time series of interest (von Asmuth et al., 2002). Different response functions can be utilized for the varying inputs of the TFN model to adequately describe the relationship to the output (von Asmuth et al., 2008; Collenteur et al., 2019). The linear TFN models applied in this study were set up with the Pastas Python package (Collenteur et al., 2019) and differ in their inputs used, with seven distinct input combinations: $\{P(t)^{BRC}\}$, $\{T(t)^{BRC}\}$, $\{Q_{riv}(t)\}$, $\{P(t)^{BRC}\& \; Q_{riv}(t)\}$, $\{T(t)^{BRC}\& \; Q_{riv}(t)\}$, $\{P(t)^{BRC}$

& $T(t)^{BRC}$}, {$P(t)^{BRC}$& $T(t)^{BRC}$& $Q_{riv}(t)$}. In a preliminary study (unpublished results of the MSc Thesis by A.O. Crespo Delgadillo) we also tested input combinations with $ET_0(t)^{BRC}$. In most cases, the model performance using T as an input feature was comparable to or even better than using ET instead. The model performance in the evaluation period was consistently better for combinations with T for all 4 wells with the RF and the TFN models. It is widely accepted that the T and ET are strongly correlated. For practical applications, air temperature is often readily available (and much easier to measure), while ET is not. For these reasons, we used T instead of ET as an input feature for the TFN models and also for the ML models described below.

For the different input types, the scaled gamma distribution was chosen as input response function for $P(t)^{BRC}$ and $T(t)^{BRC}$, while the polder response function was chosen for the $Q_{riv}(t)$ input time series. The model parameters used in our TFN model setups comprise the scaling factors $A_\Gamma \mid A_P$, the shape factors a_Γ, $n_\Gamma \mid a_P$ b_p of the gamma distribution and the polder function , respectively, as well as the base level constant d (Collenteur et al., 2019). Therefore, the parameters in the individual TFN models varies between 4, 7 or 10, depending on the number of input features utilized.

The first 80% of the total time series were used for model calibration (Table 1 of the main paper) with the standard settings of Pastas in regard to optimization options.

Multi-layer Perceptron (MLP)

For the setup of the Multi-layer perceptron (MLP) models in this study, the Python packages Keras and Tensorflow (Abadi et al., 2015) were used.

After initial testing with the input and groundwater level datasets of the wells in question, the following hyperparameters were fixed to the values given in brackets: activation function (ReLU), loss function (MSE), optimizer (ADAM), number of epochs (1) as well as performance metric for the training (MSE). In contrast, the following three hyperparameters were kept variable and optimized by performing a grid search using Keras within the distinct parameter ranges given in brackets: number of hidden layers (1 : 1 : 20), number of nodes (32 : 64 : 512), and learning rate ($1e^{-2}$,$1e^{-3}$,$1e^{-4}$).

Of the different potentially available inputs, all possible combinations of precipitation, temperature and Wairau River flow were used after preliminary testing, resulting in the same seven input combinations that were described for the TFN model. In addition, two versions of each of these combinations were used, one with window size of 60 days and one with 90 days, respectively. This resulted in 14 MLP model setups that were combined with the hyperparameter grid search.

Regarding the observation time series, most machine learning methods are more robust if data time series are scaled, especially if varying input sources are highly differing (Müller et al., 2021). For the MLP models, testing showed that standardization by mean and variance of each data time series with the Scikit-Learn Python package (Pedregosa et al., 2011) led to the best results. The overall time series were split into training and testing data equal to 80% and 20% respectively, resulting in the calibration and validation time periods listed in Table 1 of the main paper.

From these settings, the ensemble of 14 MLP models was generated by choosing the best-fitting model for each input and window size combination.

Long Short-Term Memory Networks (LSTM)

Like the MLP models, the LSTM models were set up with Keras and Tensorflow in Python.

Similarly, hyperparameters of the models were partially fixed, partially varied. Learning rate ($1e^{-3}$), activation function (ReLU), loss function (MSE), optimizer (ADAM), number of epochs (10), performance metric (MSE) and dropout rate (0.25) were fixed to the numbers in brackets, respectively. The number of hidden layers ($1 : 1 : 3$) and number of nodes ($32 : 32 : 128$) were varied within the provided ranges and best-performing combinations selected for each final LSTM model chosen.

The 14 input and window size combinations of the LSTM models were chosen to be identical to the other data-driven models as described above.

Observation time series were split into training and testing periods in identical way to the MLP model setup (Table 1 of the main paper). In contrast to the MLP model

setup, data time series for the LSTM models were scaled between 0 and 1 utilizing min-max scaling with the Scikit-Learn Python package (Pedregosa et al., 2011).

This setup resulted again in a final ensemble of of 14 chosen LSTM models for the analysis presented in the main paper.

Random Forest Model (RF)

For the Random Forest (RF) models, the Scikit-Learn Python Package (Pedregosa et al., 2011) was used for their construction and operation.

Preliminary testing led to the following settings regarding the various hyperparameters of the models: bootstrapping ($True$), maximum tree depth ($None$) and minimum number of samples for split (2) were fixed, while the number of trees (350 : 75 : 500) and the minimum number of samples per leaf (1 : 1 : 2) were chosen via grid search with the same process as described above.

The 14 input and window size combinations of the RF models were the same as for the other data-driven models presented earlier.

Time series split for training and testing periods of the observations was done identical to the other machine-learning models above, and scaling was again done using min-max scaling between 0 and 1.

Model Evaluation Criteria

The evaluation criteria applied in this study were the root mean squared error (RMSE), the coefficient of determination (R^2) and the mean bias as described in Wöhling et al. (2013), as well as the Kling-Gupta efficiency (KGE, Gupta et al., 2009). These criteria are defined as follows:

$$RMSE = \sqrt{\frac{1}{n}\sum_{i=1}^{n}\left(h_i - \hat{h_i}\right)^2}, \quad (1)$$

where h_i and $\hat{h}_i$ represent the observed and simulated groundwater levels for day i and n is the total number of days.

$$R^2 = \frac{\sum_{i=1}^{n} \left(h_i - \overline{h}\right)\left(\hat{h}_i - \overline{\hat{h}}\right)}{\sqrt{\sum_{i=1}^{n} \left(h_i - \overline{h}\right)^2 \sum_{i=1}^{n} \left(\hat{h}_i - \overline{\hat{h}}\right)^2}} \tag{2}$$

where, $\overline{h}$ is the mean of h and $\overline{\hat{h}}$ is the mean of $\hat{h}$.

$$\text{(mean)}\, Bias = \frac{1}{n} \sum_{i=1}^{n} \left(h_i - \hat{h}_i\right) \tag{3}$$

$$KGE = R^2 \left(2\alpha - \alpha^2 - \beta^2\right) \tag{4}$$

with the relative variability, α:

$$\alpha = \frac{\sum_{i=1}^{n} \left(\hat{h}_i - \overline{\hat{h}}\right)}{\sum_{i=1}^{n} \left(h_i - \overline{h}\right)} \tag{5}$$

and the normalized bias, β:

$$\beta = \frac{\overline{\hat{h}} - \overline{h}}{\sum_{i=1}^{n} \left(h_i - \overline{h}\right)}\,. \tag{6}$$

A perfect fit of the model simulations to the data would be characterized by RMSE and bias values of zero as well as R^2 and KGE values of unity.

Model residuals

The BMA scheme adopted here assumes that the model residuals, $\boldsymbol{r} = \boldsymbol{h} - \hat{\boldsymbol{h}}$, are independent and normally distributed. This assumption is convenient and rather common, but also commonly violated. We tested these assumptions for the model residuals of the best-fit model realizations of the four wells. First, we tested the residuals for normal distribution using the one-sample KS-test (Daniel, 1990) and plotted the residuals using QQ-plots. Second we tested the residuals for autocorrelation using the Ljung-Box Q-test (Box and Pierce, 1970) and plotted the Sample Partial AutoCorrelation function (PACF) using the Matlab Econometrics Toolbox. An example is depicted in

Figure 2 for well P3821.

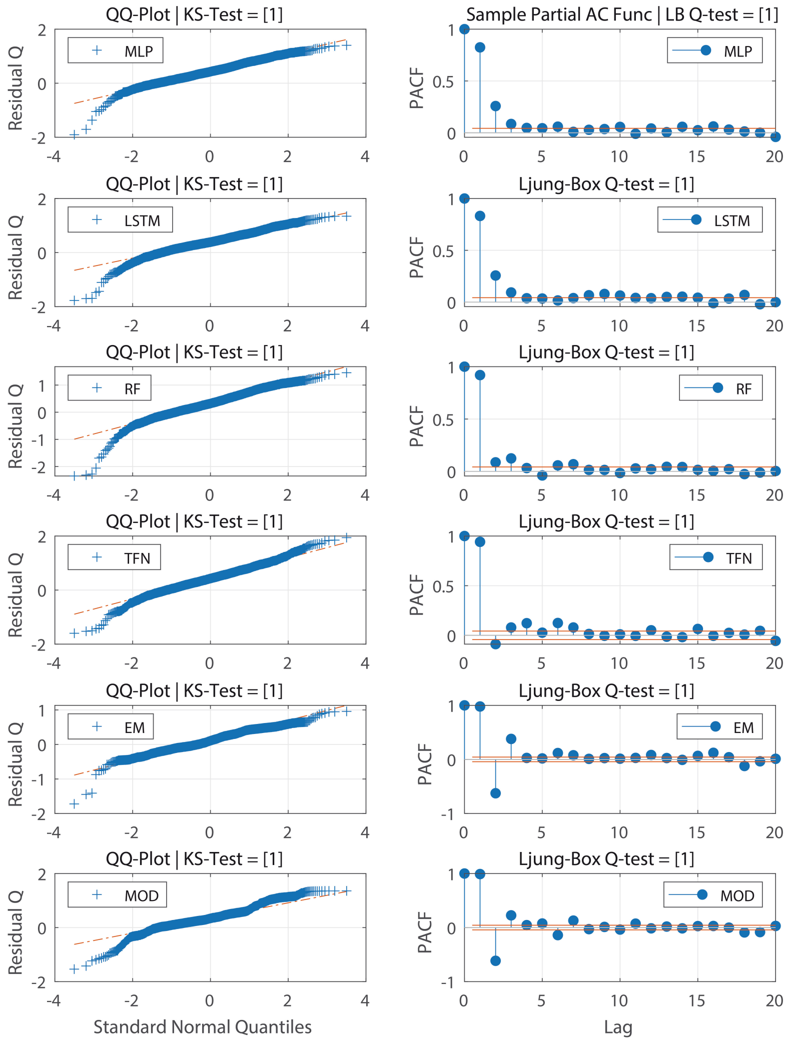

Figure 2: Model residual analysis for well P3821. The left column shows QQ-plots for the residuals of the best-fit realizations (KGE) of the six models. The right column depicts the corresponding Sample Partial AutoCorrelation functions (PACF) for these residuals. Depicted are also the results of the KS-test for normal distribution and of the Ljung-Box Q-test for autocorrelation.

Although there are differences in the individual models' distributions of residuals, in all cases, the null-hypothesis of normal distribution had to be rejected (KS = 1; at a significance level of 0.05). Similarly, the hypothesis that the residuals are not autocorrelated was rejected by the Ljung-Box Q-test in all cases. For most models, however, the autocorrelation diminishes after a lag time of one or two days. Based on these results, alternative error distribution models could be implemented and tested in future work.

References

Abadi, M., A. Agarwal, P. Barham, E. Brevdo, Z. Chen, C. Citro, G. S. Corrado, A. Davis, J. Dean, M. Devin, S. Ghemawat, I. Goodfellow, A. Harp, G. Irving, M. Isard, Y. Jia, R. Jozefowicz, L. Kaiser, M. Kudlur, J. Levenberg, D. Mané, R. Monga, S. Moore, D. Murray, C. Olah, M. Schuster, J. Shlens, B. Steiner, I. Sutskever, K. Talwar, P. Tucker, V. Vanhoucke, V. Vasudevan, F. Viégas, O. Vinyals, P. Warden, M. Wattenberg, M. Wicke, Y. Yu, and X. Zheng. 2015. TensorFlow: Large-scale machine learning on heterogeneous systems. Software available from tensorflow.org.

Bidwell, V. and L. Burbery. 2011. Groundwater Data Analysis - quantifying aquifer dynamics. Envirolink Project 420-NRLC50 Report 4110/1, Lincoln Ventures Ltd.

Bidwell, V. J. 2005. Realistic forecasting of groundwater level, based on the eigenstructure of aquifer dynamics. *Mathematics and Computers in Simulation* 69, no. 1: 12–20.

Bidwell, V. J., R. Stenger, and G. F. Barkle. 2008. Dynamic analysis of groundwater discharge and partial-area contribution to Pukemanga Stream, New Zealand. *Hydrology and Earth System Sciences* 14: 975–987.

Box, G. E. P. and D. A. Pierce. 1970. Distribution of residual autocorrelations in autoregressive-integrated moving average time series models. *Journal of the American Statistical Association* 65, no. 332: 1509–1526.

Brown, L. J. 1981. Late Quaternary geology of the Wairau Plain, Marlborough, New Zealand. *New Zealand Journal of Geology and Geophysics* 24: 477–490.

Collenteur, R. A., M. Bakker, R. Caljé, S. A. Klop, and F. Schaars. 2019. Pastas: Open Source Software for the Analysis of Groundwater Time Series. *Groundwater* 57, no. 6: 877–885.

Daniel, W. W. 1990. *"Applied Nonparametric Statistics"*, chapter "Kolmogorov-Smirnov one-sample test", pages 319–330. Duxbury classic series. PWS-Kent., Boston, 2nd edition.

Doherty, J. 2003. Ground water model calibration using pilot points and regularization. *Groundwater* 41, no. 2: 170–177.

Doherty, J. E. 2016a. *PEST, Model-independent parameter estimation, user manual Part I.* Watermark Numerical Computing, Brisbane, Australia, 6th edition.

Doherty, J. E. 2016b. *PEST, Model-independent parameter estimation user manual Part II: PEST utility support software.* Watermark Numerical Computing, Brisbane, Australia, 6th edition.

Gupta, H. V., H. Kling, K. K. Yilmaz, and G. F. Martinez. 2009. Decomposition of the mean squared error and NSE performance criteria: Implications for improving hydrological modelling. *Journal of Hydrology* 377, no. 1-2: 80–91.

Harbaugh, A. 2005. Modflow-2005, the u.s. geological survey modular ground-water model - the ground-water flow process:. U.S. Geological Survey Techniques and Methods 6-A16., U.S. Geological Survey.

Minhas, B. S., K. S. Parikh, and T. N. Srinivasan. 1974. Toward the structure of a production function for wheat yields with dated inputs of irrigation water. *Water Resour. Res.* 10, no. 3: 383–393.

Müller, J., J. Park, R. Sahu, C. Varadharajan, B. Arora, B. Faybishenko, and D. Agarwal. 2021. Surrogate optimization of deep neural networks for groundwater predictions. *Journal of Global Optimization* 81, no. 1: 203–231.

Niswonger, R., S. Panday, and M. Ibaraki. 2011. MODFLOW-NWT, A Newton formulation for MODFLOW-2005. Techniques and Methods 6-A37, U.S. Geological Survey. 44 p.

Niswonger, R. G. and D. E. Prudic. 2005. Documentation of the Streamflow-Routing (SFR2) Package to include unsaturated flow beneath streams–A modification to SFR1. Techniques and Methods 6-A13, U.S. Geological Survey.

Pedregosa, F., G. Varoquaux, A. Gramfort, V. Michel, B. Thirion, O. Grisel, M. Blondel, P. Prettenhofer, R. Weiss, V. Dubourg, J. Vanderplas, A. Passos, D.

Cournapeau, M. Brucher, M. Perrot, and E. Duchesnay. 2011. Scikit-learn: Machine learning in Python. *Journal of Machine Learning Research* 12: 2825–2830.

Pondard, N. and P. M. Barnes. 2010. Structure and paleoearthquake records of active submarine faults, cook strait, new zealand: Implications for fault interactions, stress loading, and seismic hazard. *Journal of Geophysical Research: Solid Earth* 115, no. B12.

Pulido-Velazquez, D., D. Ahlfeld, J. Andreu, and A. Sahuquillo. 2008. Reducing the computational cost of unconfined groundwater flow in conjunctive-use models at basin scale assuming linear behaviour: The case of Adra-Campo de Dalias. *Journal of Hydrology* 353, no. 1: 159–174.

Pulido-Velazquez, D., A. Sahuquillo, and J. Andreu. 2011. A conceptual-numerical model to simulate hydraulic head in aquifers that are hydraulically connected to surface water bodies. *Hydrological Processes* 26, no. 10: 1435–1448.

Pulido-Velazquez, D., A. Sahuquillo, J. Andreu, and M. Pulido-Velazquez. 2007a. An efficient conceptual model to simulate surface water body-aquifer interaction in conjunctive use management models. *Water Resources Research* 43, no. 7.

Pulido-Velazquez, D., A. Sahuquillo, J. Andreu, and M. Pulido-Velazquez. 2007b. A general methodology to simulate groundwater flow of unconfined aquifers with a reduced computational cost. *Journal of Hydrology* 338, no. 1: 42–56.

Pulido-Velazquez, M. A., A. Sahuquillo-Herraiz, J. Camilo Ochoa-Rivera, and D. Pulido-Velazquez. 2005. Modeling of stream-aquifer interaction: the embedded multireservoir model. *Journal of Hydrology* 313, no. 3: 166–181.

Sahuquillo, A. 1983. An eigenvalue numerical technique for solving unsteady linear groundwater models continuously in time. *Water Resources Research* 19, no. 1: 87–93.

von Asmuth, J., M. Bierkens, and K. Maas. 2002. Transfer function-noise modeling

in continuous time using predefined impulse response functions. *Water Resources Research* 38, no. 12.

von Asmuth, J. R., K. Maas, M. Bakker, and J. Petersen. 2008. Modeling time series of ground water head fluctuations subjected to multiple stresses. *Groundwater* 46, no. 1: 30–40.

Wilson, S. R. 2016. Wairau Aquifer stratigraphy review. Technical Report 1053-1-R1, Lincoln Agritech Ltd.

Wöhling, T. 2019. Natürliche und anthropogene Einflussfaktoren auf das hydrologische Regime des Wairau Plain Aquifer in Neuseeland. *Hydrologie und Wasserbewirtschaftung* 63, no. H3: 130–140. (in German).

Wöhling, T. and L. Burbery. 2020. Eigenmodels to forecast groundwater levels in unconfined river-fed aquifers during flow recession. *Science of The Total Environment* 747: 141220.

Wöhling, T., M. J. Gosses, S. R. Wilson, and P. Davidson. 2018. Quantifying river-groundwater interactions of New Zealand's gravel-bed rivers: The Wairau Plain. *Groundwater* 56, no. 4: 647–666.

Wöhling, T., L. Samaniego, and R. Kumar. 2013. Evaluating multiple performance criteria to calibrate the distributed hydrological model of the upper neckar catchment. *Environmental Earth Sciences* 69, no. 2: 453–468. Special Issue on Catchment Research.

Wöhling, T., S. Wilson, V. Wadsworth, and P. Davidson. 2020. Detecting the cause of change using uncertain data: Natural and anthropogenic factors contributing to declining groundwater levels and flows of the Wairau Plain aquifer, New Zealand. *Journal of Hydrology: Regional Studies* 31, no. 10: 100715.
